# Xanthone Glucosides: Isolation, Bioactivity and Synthesis

**DOI:** 10.3390/molecules26185575

**Published:** 2021-09-14

**Authors:** Qing Huang, Youyi Wang, Huaimo Wu, Man Yuan, Changwu Zheng, Hongxi Xu

**Affiliations:** 1 School of Pharmacy, Shanghai University of Traditional Chinese Medicine, Shanghai 201203, China; huangqingwork@126.com (Q.H.); 17317580739@163.com (Y.W.); 15709616889@163.com (H.W.); peggyyuan1990@163.com (M.Y.); 2 Engineering Research Center of Shanghai Colleges for TCM New Drug Discovery, Shanghai 201203, China

**Keywords:** xanthone *C*-glucoside, xanthone *O*-glucoside, chemical synthesis, pharmacological activity, 9*H*-xanthen-9-one

## Abstract

Xanthones are secondary metabolites found in plants, fungi, lichens, and bacteria from a variety of families and genera, with the majority found in the Gentianaceae, Polygalaceae, and Clusiaceae. They have a diverse range of bioactivities, including anti-oxidant, anti-bacterial, anti-malarial, anti-tuberculosis, and cytotoxic properties. Xanthone glucosides are a significant branch of xanthones. After glycosylation, xanthones may have improved characteristics (such as solubility and pharmacological activity). Currently, no critical review of xanthone glucosides has been published. A literature survey including reports of naturally occurring xanthone glucosides is included in this review. The isolation, structure, bioactivity, and synthesis of these compounds were all explored in depth.

## 1. Introduction

In natural product chemistry, xanthones are one of the most abundant types of chemicals. They are secondary metabolites found in higher plant families, fungi, lichen, and bacteria, and are primarily found in Gentianaceae, Polygalaceae, Clusiaceae, and others [1,2,3]. They have a variety of health-promoting properties, including anti-bacterial, anti-carcinogenic, anti-oxidant, and anti-diabetic properties [4,5,6,7,8].

The structure of xanthone determines its bioactivity, and different substitutions might result in a variable bioactivity [9,10,11]. The chemical formula of xanthone is C_13_H_8_O_2_. Its main structure is 9*H*-xanthen-9-one with a dibenzo-*γ*-pirone scaffold. Research on xanthones has received much attention in recent years [12,13,14,15]. In general, xanthones are categorized into six classes based on substitutions on the basic structure of xanthones: simple xanthones, xanthone glucosides (or glycosylated xanthones), prenylated xanthones, xanthonolignoids, bis-xanthones, and miscellaneous xanthones [16,17]. The main distribution of these xanthones varies, as prenylated xanthones are widely distributed in the Clusiaceae and most compounds of simple xanthones and xanthone glucosides are from the Gentianaceae. These primary groupings are further subdivided into non, mono-, di-, tri-, tetra-, penta-, hexa-, and hepta-oxygenated xanthones based on the degree of oxygenation [18,19,20].


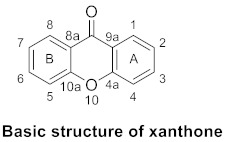


More recently, xanthone glucosides have been explored, and the mutation of these glycosyl groups can change the biological activity of xanthone, which has a wide range of clinical applications [21,22]. However, xanthones usually have poor solubility; herein, many studies are being devoted to the synthesis of glycosylated xanthones to improve their solubility and activity and minimize their toxicity [23,24]. Xanthone glucosides are an important class of xanthones that are extensively dispersed in the plant families Gentianaceae and Polygalaceae. For natural xanthone glucosides, each xanthone site can be connected to a sugar group, which can be either monosaccharide or disaccharide. Recent research has revealed that xanthone glucosides have anti-oxidant [25], anti-inflammatory [26], anti-cancer [21,27], and other pharmacological properties. We separated xanthone glucosides into xanthone *C*-glucoside and xanthone *O*-glucoside and classified the substances accordingly. *C*–*C* bonds connect the sugar moiety to the xanthone nucleus in *C*-glucosides, which are usually resistant to acidic and enzymatic hydrolysis, whereas *O*-glucosides have normal glycosidic linkages. In glucosides whose glycosyl group is disaccharide, the second sugar residue is often glucose, xylose, or rhamnose and is usually associated with *C*-6 of the first glucose unit. However, when the second residue is rhamnose, it is linked to the *C*-2 of the first residue. The structures and connection site of sugars to the xanthone core that may be used in their full names are shown below.


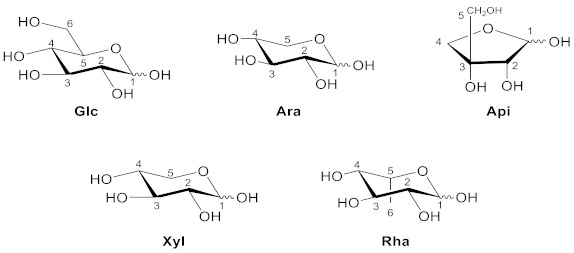


In general, xanthone glucosides have received much interest due to their unique structures and significant bioactivities. As a result, we examined the separation, bioactivity, and synthesis of naturally occurring xanthone glucosides, with the goal of providing a reference for future relevant studies.

## 2. Structure, Isolation and Bioactivity of Xanthone Glucoside

### 2.1. Xanthone C-Glucoside

This class of xanthone glucosides is composed of xanthone and sugar groups that are linked together by carbon atoms in the structure. D-glucose is a sugar group that is commonly found in these compounds. The majority of the sugar binding sites are located at position 2, and glycosylation can often boost the activity to a certain amount [28]. All of the xanthones have hydroxyl substitutions on their skeletons, and some of them have methoxy groups. The scavenging of free radicals and the anti-oxidant activity of these compounds are their most notable impacts. We will classify these compounds by distinct genera in the order in which they were discovered, followed by a description of their biological activity.

#### 2.1.1. Xanthone *C*-Glucoside from Liliaceae

Mangiferin (**1**) is the most widely studied xanthone *C*-glucoside for pharmaceutical purposes [29,30], and it may be obtained from a variety of plants, including *Anemarrhena asphodeloides* Bge (Liliaceae) [31], *A. senkakuinsulare* (Aristolochiaceae) [32], *Mahkota dewa* (Phaleria macrocarpa (Scheff.) Boerl) [33], *Coffea pseudozanguebariae* (*Rubiaceae*) [34], and *Lomatogonium carinthiacum* (Gentianaceae) [35]. There is a glucose substitution at position 2 of the xanthone skeleton in mangiferin, as well as hydroxyl substitutions at positions 1, 3, 6, and 7. Mangiferin’s *C*-glycosidic bond, which mimics the nucleophilic substitution of phloroglucinol, improves bio-availability and is responsible for its anti-oxidant properties [36]. Mangiferin has been shown to have anti-inflammatory activity [37,38,39], anti-oxidant activity [40,41,42], anti-diabetic activity [43,44,45], cardio-protective effects [46,47,48], and anti-cancer activity [49,50,51]. The anti-inflammatory and anti-oxidant activities were due to the free radical scavenging capacity of mangiferin [41,52,53]. Mangiferin is a potent inhibitor of the NF-Kappa B signaling pathway [54], and the anti-oxidant activity of mangiferin is also related to its iron-chelating properties [55].


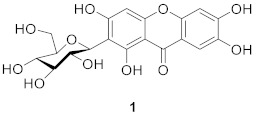


In 1970, Aritomi and Kawasaki isolated homomangiferin (**2**) and isomangiferin (**3**) from *Anemarrhena asphodeloides* Bunge [56]. These two compounds were similar to mangiferin in structure. Homomangiferin has a methoxy group at position 3 compared to mangiferin, while the sugar group of isomangiferin is attached at position 4. Isomangiferin can also be isolated from *Cyclopia genistoides* (L.) Vent. (honeybush) and has a strong effect in the treatment of rheumatoid arthritis [57].

In 1997, Guo’s team isolated neomangiferin (**4**) from *Anemarrhena asphodeloides* Bge. The structure of the compound was 7-*O*-*β*-d-glucopyranosyl-mangiferin [31], which thus far is the only xanthone glucoside that contains both *C*-glucoside and *O*-glucoside. Neomangiferin was found to modulate the Th17/Treg balance and ameliorate colitis in mice [58].


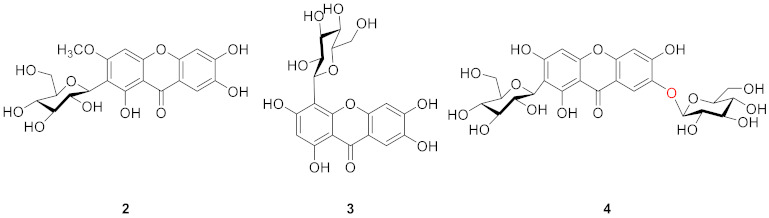


#### 2.1.2. Xanthone *C*-Glucoside from Iridaceae

In 1973, Takemoto’s team isolated irisxanthone (**5**) from *Iris florentina* L. Compared to mangiferin, irisxanthone has a methoxy group at the 5-position and no hydroxyl group at the 7-position [59]. Irisxanthone can also be isolated from the leaves of *I. albicans* Lange [60], *Iris adriatica* [61], and *Iris germanica* [62]. In 1995, Alkhalil’s team isolated 2-*β*-d-glucopyranosyl-l,3,5,8-tetrahydroxyxanthone (nigricanside) (**6**) from the rhizomes of *Iris nigricans*, and the structure of the compound was 2-*β*-d-glucopyranosyl-l,3,5,8-tetrahydroxy-9*H*-xanthene-9-one [63]. 


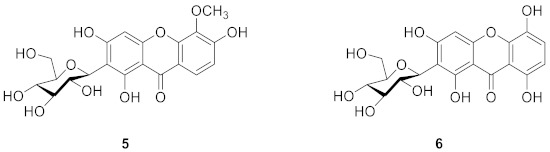


#### 2.1.3. Xanthone *C*-Glucoside from Arrabidaea

In 2003, Bolzani’s team isolated 2-(2′-*O*-*trans*-caffeoyl)-*C*-*β*-d-glucopyranosyl-1,3,6,7-tetrahydroxyxanthone (**7**), 2-(2′-*O*-*trans*-cinnamoyl)-*C*-*β*-d-glucopyranosyl-1,3,6,7-tetrahydroxyxanthone (**8**), and 2-(2′-*O*-*trans*-coumaroyl)-*C*-*β*-d-glucopyranosyl-1,3,6,7-tetrahydroxyxanthone (**9**) from the stems of *Arrabidaea samydoides*. These compounds showed moderate free radical scavenging activity against 1,1-diphenyl-2-picrylhydrazyl (DPPH) [64]. In 2008, Hostettmann’s team isolated 3′-*O*-*p*-hydroxybenzoylmangiferin (**10**), 3′-*O*-*trans*-coumaroylmangiferin (**11**), 6′-*O*-*trans*-coumaroylmangiferin (**12**), 3′-*O*-*trans*-cinnamoylmangiferin (**13**), 3′-*O*-*trans*-caffeoylmangiferin (**14**), and 3′-*O*-benzoylmangiferin (**15**) from the leaves of *Arrabidaea patellifera*. Compounds **10**–**12** have demonstrated anti-plasmodial activity (IC_50_: 26.5 μM, 18.1 μM and 38.2 μM, respectively). In addition, compounds **10**–**15** (shown in Table 1) have shown radical-scavenging and anti-oxidant activities [65]. 


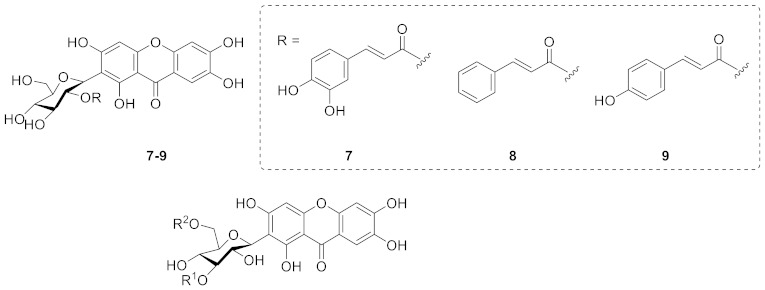


#### 2.1.4. Xanthone *C*-Glucoside from Polygalaceae

In 1999, sibiricaxanthones A (**16**) and sibiricaxanthones B (**17**) were isolated from the roots of *Polygala sibirica* by Miyase’s group. The structure of **16** was 2-*C*-[*β*-d-apiofuranosyl-(1→6)-*β*-d-glucopyranosyl]-1,3,7-trihydroxyxanthone, and **17** was shown to be 2-*C*-[*β*-d-apiofuranosyl-(1→2)-*β*-d-glucopyranosyl]-1,3,7-trihydroxyxanthone [66]. In 2005, Tu’s group isolated polygalaxanthones VIII (**18**) and polygalaxanthones XI (**19**) from the cortexes of *Polygala tenuifolia*. The structure of **18** was defined as 2-*C*-[*β*-d-arabinopyranosyl-(1→6)-*β*-d-glucopyranosyl]-1,3,7-trihydroxy-6-methoxyxanthone and **19** was 2-C-[*β*-d-apiofuranosyl-(1→2)-*β*-d-glucopyranosyl]-1,3,7-trihydroxy-6-methoxyxanthone [67].

Telephioxanthones A (**20**) and Telephioxanthones B (**21**) are two xanthone *C*-glucosides isolated from *Polygala telephioides* by Tu’s group in 2007. Compound **20** was shown to be 6’-*O*-[(E)-cinnamoyl]mangiferin), and compound **21** was 4’-*O*-[(E)-cinnamoyl]mangiferin [68]. Polygalaxanthone III (**22**), is a xanthone glucoside isolated from polygala root [69] that showed a potential scavenging effect on DPPH and hydroxy radicals and reductive activity to Fe^3+^ with IC_50_ values of 76.1, 83.5, and 54.9 mM [70]. 

In 2013, a new xanthone *C*-glucoside, tenuiside A (**23**), along with three known xanthone *C*-glucosides, lancerin (**24**) [71], neolancerin (**25**) [72], and 7-*O*-methylmangiferin (**26**) [73] were isolated from *Polygala tenuifolia* by Jiang’s group. Compounds **23**–**25** have NO inhibitory activity and low cytotoxicity. Compound **24** showed stronger activity than compound **25**, indicating that glycosidation at *C*-4 is superior to glycosidation at *C*-2 in terms of inhibition of NO [74].


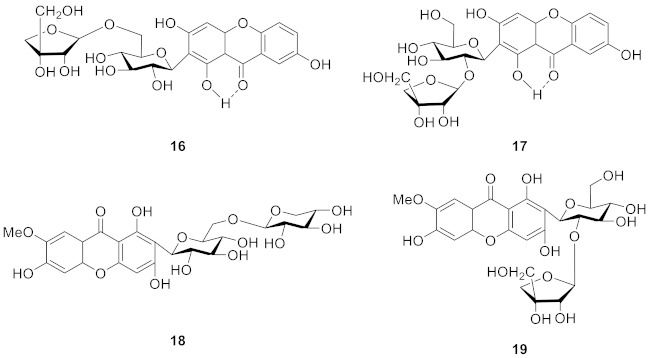



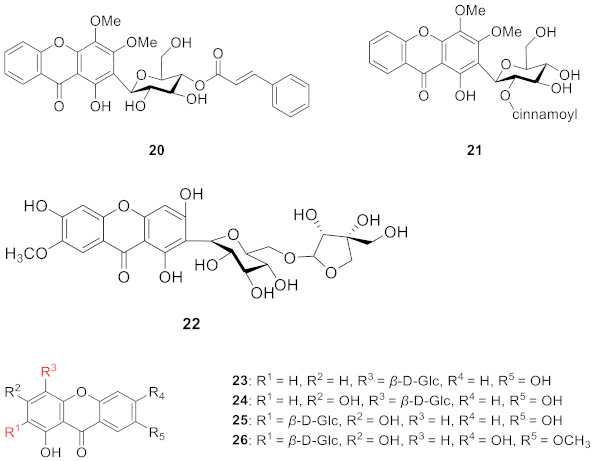


#### 2.1.5. Xanthone *C*-Glucoside from Gentianaceae

In 1991, Cordell’s team isolated swertipunicosid (**27**) from *Swertia punicea* Hemal., which was the first bisxanthone *C*-glucoside. The structure was 1,5,8-trihydroxy-3-methoxy-7-(1′,3′,6′,7′-tetrahydroxy-9′-oxo-4′-xanthyl) xanthone 2′-*C*-*β*-d-glucopyranoside [75]. In 1992, the same team isolated 3-*O*-demethylswertipunicoside (**28**) from *Swertia punicea*, the structure of which was 1,3,5,8-tetrahydroxy-7-(1′,3′,6′,7’-tetrahydroxy-9′-oxo-4′-xanthyl) xanthone 2′-*C*-*β*-d-glucopyranoside [76]. According to later research, the compound **28** showed potent neuro-protective activity against H_2_O_2_-induced PC12 cell damage [77].

In 2010, Guo’s team isolated two new xanthone *C*-glucosides, puniceaside D (**29**) and puniceaside E (**30**), from *Swertia punicea*. Puniceasides D and E are two unique trimeric xanthone *C*-glucosides [77]. In 2013, 3,5,6,8-tetrahydroxyxanthone-1-*C*-*β*-d-glucoside (**31**), which has excellent anti-oxidant activity, was isolated from Swertia mussotii. According to the research, glycosylated xanthones are more active than those that are not [78]. In 2016, Zhang’s team isolated apigenin-7-*O*-gluco (1″→3‴) glucoside (**32**) from *Gentianella turkestanorum*, the structure of which is similar to mangiferin, with a hydroxyl group missing at the 7-position [79].


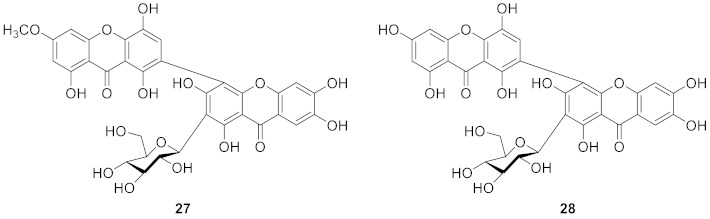



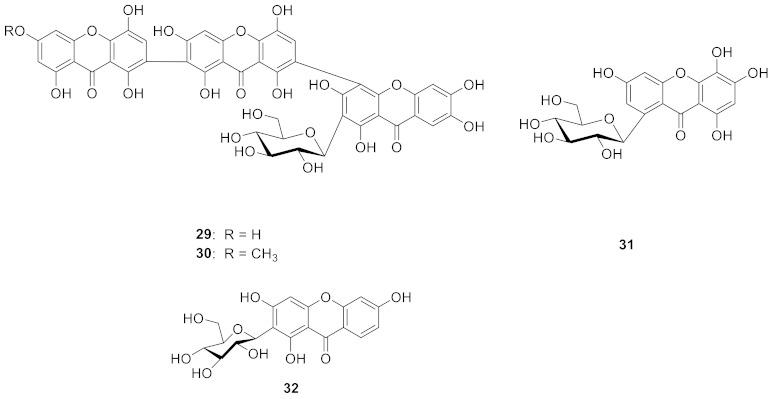


#### 2.1.6. Xanthone *C*-Glucoside from Bombacaceae

Shamimoside (**33**) was isolated from the leaves of *Bombax ceiba* L. by Versiani’s team. The structure of the compound was 4-*C*-*β*-d-glucopyranosyl-1,3,6,8-tetrahydroxy-7-*O*-(p-hydroxybenzoyl)-9*H*-xanthen-9-one and it is the first naturally occurring xanthone containing a benzoate moiety directly attached to an aromatic ring. The DPPH anti-oxidant assay shows that the compound has moderate anti-oxidant activity (IC_50_ = 150 μg/mL) [29]. 


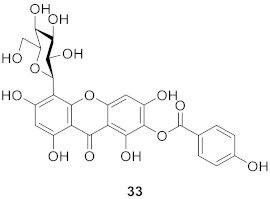


#### 2.1.7. Others

Mangiferoxanthone A (**34**) is a xanthone dimer isolated from *M. indica* by bioassay in 2014, and is a symmetric homodimer of mangiferin. The compound showed moderate influenza neuraminidase inhibition activity. According to the research, dimerization increased the activity of the compound compared with mangiferin [80]. 


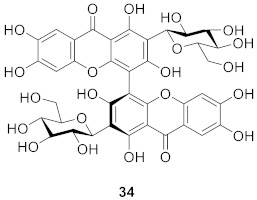


### 2.2. Xanthone O-Glucoside

In contrast to xanthone *C*-glucosides, xanthone *O*-glucosides are glucosides that are linked to the tricyclic body of xanthones by an oxygen atom. Xanthone glucoside is generally found at the *C*-1 position of the xanthone nucleus. Glucosides are typically monosaccharides or disaccharides that contain glucose, xylose, rhamnose, and other glycosyl groups. At present, most xanthone *O*-glucosides isolated from natural resources contain hydroxyl, and methoxy groups, and a few have methyl groups, aliphatic side chains, or aromatic rings. Xanthone *O*-glucosides, in general, are a well-studied class of compounds. The glycosylation of xanthones improves not only their physical properties (such as solubility) but also their biological activity.

#### 2.2.1. Xanthone *O*-Glucoside from Gentianaceae

In 1969, Stout and Balkenhol identified a xanthone *O*-glucoside whose structure is 1-(*β*-d-glucosyloxy)-8-hydroxy-3,5-dimethoxyxanthone (**35**) from the root of *Frasera carolinicnsis* Walt [81].


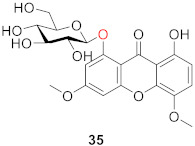


In the same year, Tomimori and Komatsu obtained norswertianolin (**36**) from *Swertia macrosperma* for the first time [82]. Four years later, Tomimori’s team discovered a new xanthone *O*-glucoside in *Swertia* spp., named norswertianin-1-glucoside (**37**) [83].


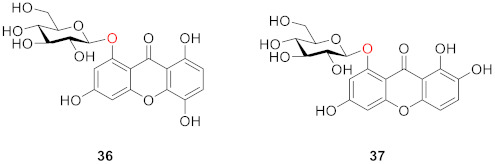


Four compounds (**38**–**41**) were isolated from *Gentiana bavarica* L. by Hostettmann’s group in 1974: gentiabavaroside (**38**), gentiabavarutinoside (**39**), isogentiakochianoside (**40**), and norswertiaprimevdroside (**41**). Structurally, they all contain disaccharide substituents, with the other substituents being hydroxyl or methoxyl groups, respectively [84].


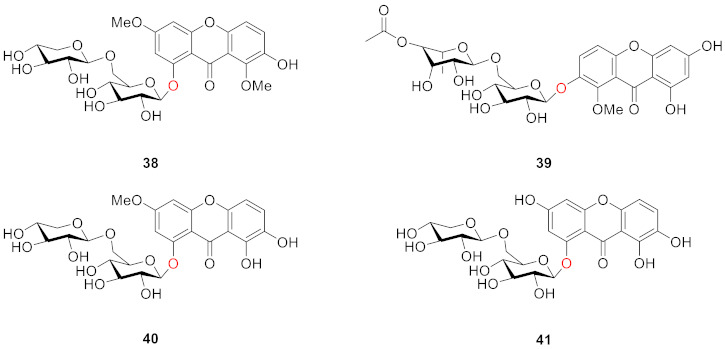


In 1977, a new xanthone diglucoside (**42**) was isolated from the aerial parts of *Swertia perennis* L. (Gentianaceae) by means of column chromatography on polyamide, followed by preparative TLC. Its structure has been established as 1,3-di-*β*-d-glucopyranosyl-7,8-dihydroxyxanthone or norswertianine-1,3-diglucoside [85].


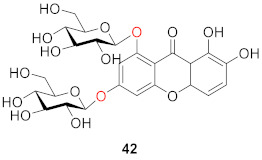


In 1978, Ghosal extracted and isolated five compounds (**43**–**47**) that had not been reported before from *Swertia angustifoh* Buch.-Ham. Their study showed that xanthone *O*-glucosides in the plant could be identified after the onset of maturity (i.e., 4- to 6-week-old plants) and were not present at the beginning of growth [86].


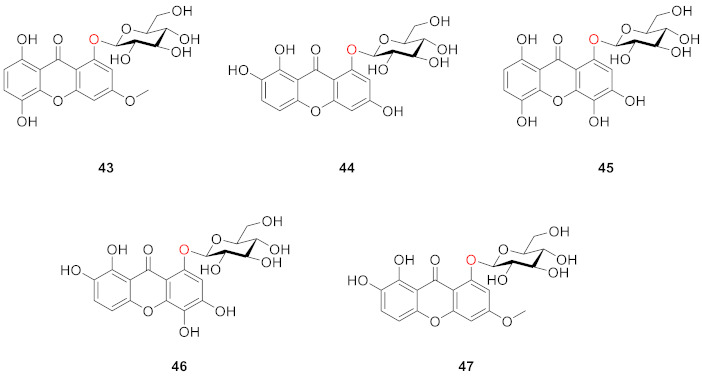


Dhasmana and Garg isolated 2,3,7-trimethoxyxanthone-1-*O*-glucoside (**48**) and 2,3,5-trimethoxyxanthone-1-*O*-glucoside (**49**) from *Halenia elliptrca* D. Don. in 1989, and parts from an alcoholic plant extract containing these two compounds showed anti-amoebic activity. The structural difference between these two compounds is that the methoxy groups are at sites 2, 3, and 7 in **48** and 2,3, and 5 in **49** [87].


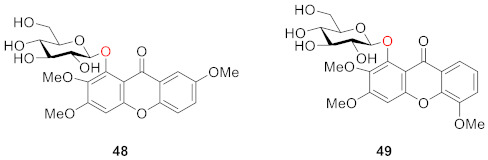


After extraction and analysis, Sun’s group obtained three compounds from *Swertia mussotii* Franch. in 1991, namely 7-*O*-*β*-d-xylopyranosyl-1,8-dihydroxy-3-methoxyxanthone (**50**), 7-*O*-[*α*-ʟ-rhanopyranosyl-(1–2)-*β*-d-xylopyranosyl]-1,8-dihydroxy-3-methoxyxanthone (**51**), and 3-*O*-*β*-d-glucopyranosy-1,8-dihydroxy-5-methoxyxanthone (**52**). They all have hydroxyl groups at positions 1 and 8. These three compounds were isolated from water-soluble components, demonstrating that the glycosyl group in the structure was the main factor influencing their solubility [88].


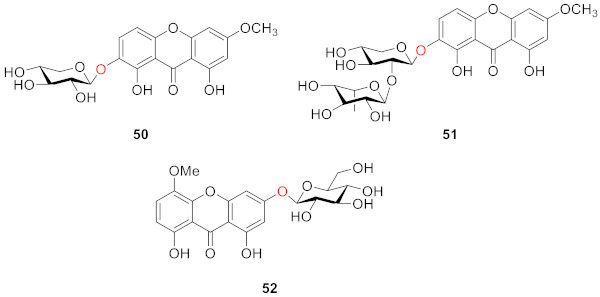


Hosteyitman and coworkers discovered and identified three compounds in 1992: 2,3,5-trimethoxy-1-*O*-gentiobiosyloxyxanthone (**53**), 2,3,5-trimethoxy-l-*O*-primeverosyloxyxanthone (**54**), and 2,3,4,5-tetramethoxy-1-*O*-primeverosyloxyxanthone (**55**). The other substituents of these three compounds, such as those of compounds **48** and **49**, are all methoxy groups, and their glycosidic bond is at the 1-position. These three compounds, however, are disaccharide substituted, in contrast to the former [89].


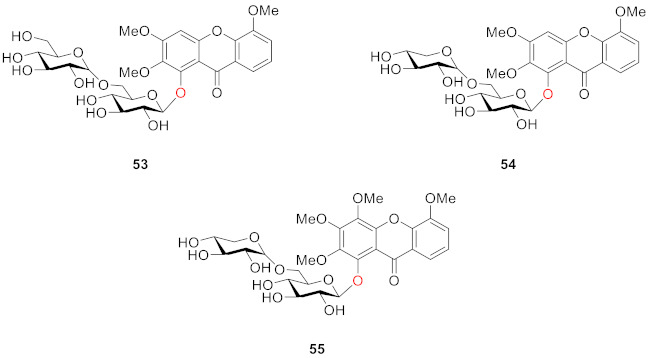


In 1995, Hostettmann’s group isolated and identified eight xanthone *O*-glucosides (**56**–**63,** shown in Table 2) from *Halenia corniculata*. These eight compounds share the following characteristics: (1) they all have three or four methoxy groups, and (2) they are disaccharides with gentiobiose or primeverose at the *C*-1 position. Their structures are similar to those discovered by Hosteyitman (**53**–**55**) [90]. 


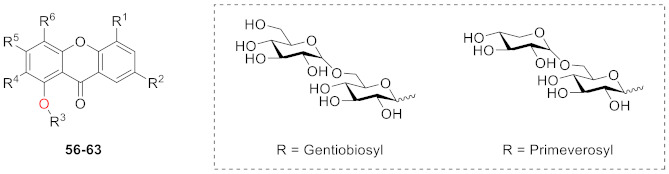


From the aerial parts of *Tripterospermum japonicum*, five new xanthone glucosides, named triptexanthosides A-E (**64**–**68**), were isolated along with a known xanthone *C*-glucoside, mangiferin, by Hideaki Ostuka in 1999. Their structures were elucidated as 1,2,6,8-tetrahydroxyxanthone 1-*O*-*β*-d-glucopyranoside (**64**), 1-*O*-*β*-gentiobioside (**65**), 1,2,8-trihydroxy-5,6-dimethoxyxanthone 2-*O*-*β*-d-glucopyranoside (**66**), 2-*O*-*β*-primeveroside (**67**), and 1-*O*-gentiobioside (**68**) [91].


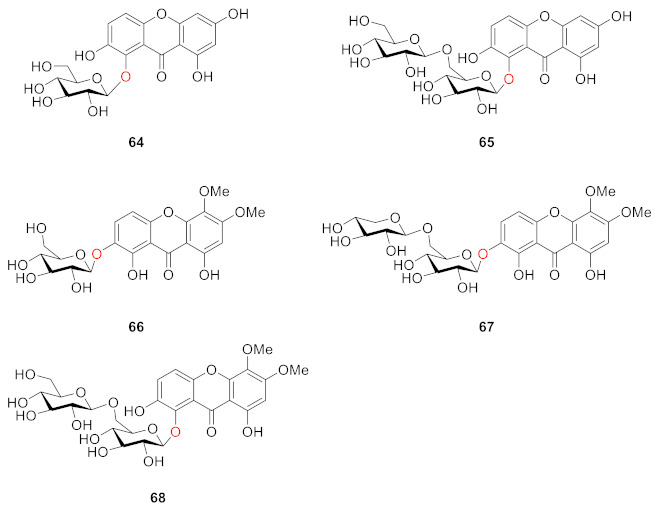


Milosavljević isolated two compounds, 1-*O*-primeverosyl-3,8-dihydroxy-5-methoxyxanthone (**69**) and 1-*O*-gentiobiosyl-3,7-dimethoxy-8-hydroxyxanthone (**70**), from another plant of the Gentianaceae (*Swertia punctate*) in 2002. They have disaccharide substituents such as gentiobiosyl and primeverosyl, which are the same as the substituents of the compound discovered in **56**–**63** [92].


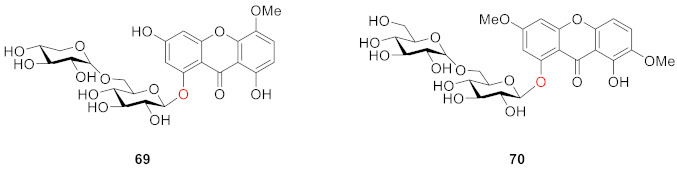


Tan and colleagues isolated a xanthone *O*-glucoside, 5-*O*-*β*-d-glucopyranosyl-1,3,8-trihydroxyxanthone (**71**), from *Swertia davidii* Franch in 2004 [93]. Meanwhile, 8-*O*-*β*-d-glucopyranosyl-1,3,5-trihydroxyxanthone (norswertianolin, **72**, isomers of **71** and **64**), discovered with 1,5-dihydroxy-3-methoxyxanthone-8-*O*-*β*-d-glucopyranoside (**73**) from *Gentiana campestris* by Kaldas and co-workers in 1974 [94], was isolated for the first time from *Swertia davidii* Franch. by the same group [93]. 
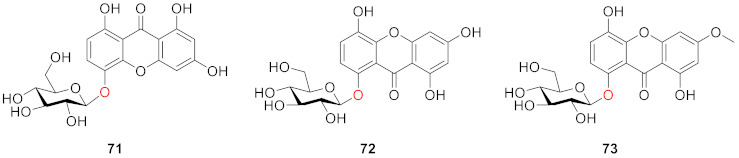


In 2005, Rana’s team isolated 6-hydroxy-3,5-dimethoxy-1-[(6-*O*-*β*-d-xylopyranosyl-*β*-d-glucopyranosyl)oxy]-9*H*-xanthen-9-one (**74**) from the rhizomes of *Swertia speciosa*, and the compound had moderate 2,2-di(4-tert-octylphenyl)-1-picryl-hydrazyl (DPPH) radical scavenging activity [95].


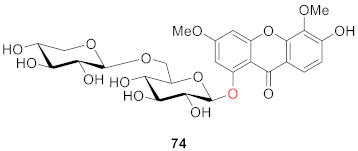


One year later, from the aerial parts of *Swertia longifolia* Boiss., which grows in northern of Iran, two diglycosidic xanthones were isolated. The structures were confirmed by means of their spectral data as 1,5-dihydroxy-3-methoxy-6-*O*-primeverosyl xanthone (**75**) and 8-hydroxy-3,5-dimethoxy-1-*O*-primeverosyl xanthone (**76**), which are new derivatives in the plant kingdom [96].


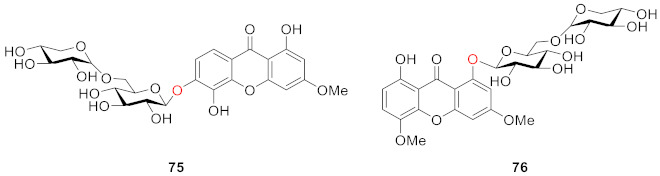


In 2008, corymbiferin 3-*O*-β-d-glucopyranoside (**77**) and swertiabisxanthone-I 8′-*O*-*β*-d-glucopyranoside (**78**) were isolated and identified from *Gentianella amarella* ssp. acuta (Michx.) J.M.Gillett by Hostettmann, in which **78** was a dimer of xanthones. Moreover, these two compounds also showed a weak inhibitory effect on acetylcholinesterase (AChE) and monoamine oxidases (MAO) A and B, which are associated with Alzheimer’s disease [97,98,99]. The inhibitory rates of **77** on MAO A and B were 40.2 ± 2.6% and 47.8 ± 2.2%, respectively. The inhibitory rates of **78** to MAO A and B were 21.4 ± 4.9% and 39.1 ± 1.2%, respectively [100].


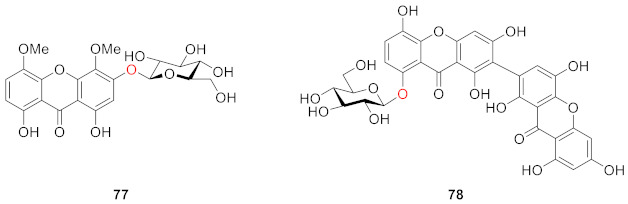


In 2010, two new dimeric xanthone *O*-glucosides, puniceasides A (**79**) and B (**80**), a new trimeric *O*-glucoside, puniceaside C (**81**), and a known xanthone *O*-glucoside swertiabisxanthone-I 8′-*O*-*β*-d-glucopyranoside (**82**) were isolated from the entire plant of *Swertia punicea*. The compounds were evaluated for their potential neuroprotective activities against H_2_O_2_-induced PC12 cell damage using an MTT assay. Compounds **80** and **82** displayed potent neuroprotective activity [77].


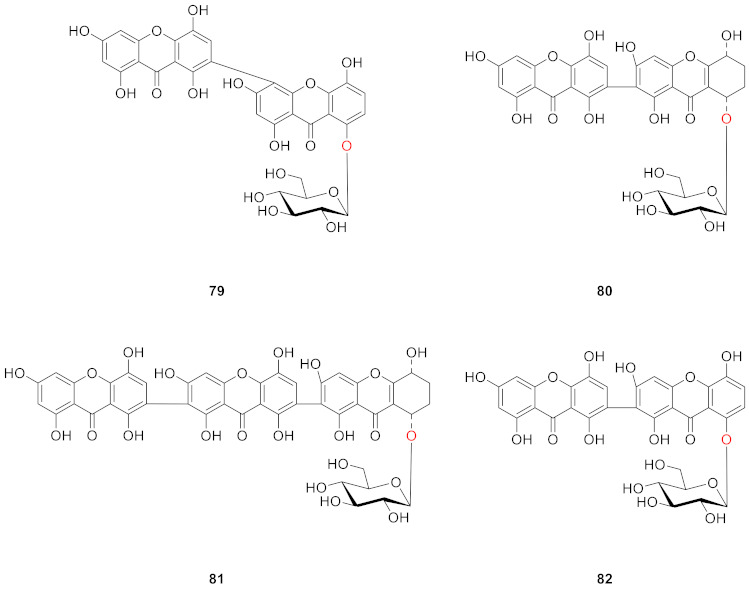


Feng and co-workers found comastomaside A (**83**) in *Comastoma pedunlulatum* (Rogle eX D. Dou) Holub in 2011, which is a traditional Tibetan medicine named Zangyinchen. Structurally, **83** is different from other compounds in that it has an aryl side chain on its glucoside chain [101].


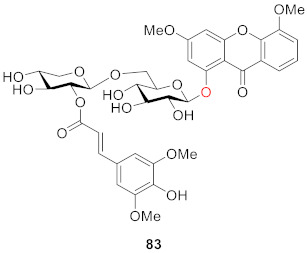


In 2011, Ding’s group conducted several activity tests on 1-*O*-*β*-d-glucoside-7-hydroxyl-3,8-dimethoxyxanthone (**84**) from *Gentianopsis paludosa* Ma. The compound exhibited significant cytotoxicity, with IC_50_ values of 18.00 ± 0.84 (μg/mL) in HepG2 cells and 24.80 ± 1.79 (μg/mL) in HL-60 cells. At the same time, the compound can inhibit cell proliferation while inducing apoptosis in both types of cells [102].


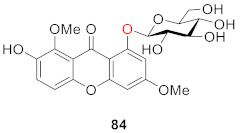


Li’s group found that 1-*O*-*β*-d-glucopyranosyl-3,5,6-trimethoxy-xanthone (**85**) from *Swertia mussotii* Franch. had a weak inhibitory effect on *α*-glucosidase, with an inhibition rate of 5.4% when the concentration was 40 μM. The other compound 1-*O*-[*β*-d-xylopyranosyl-(1→6)-*β*-d-glucopyranosyl]-3,5,6-trimethoxy-xanthone (**86**) from *Swertia mussotii* Franch had a weak inhibitory effect on dipeptidyl peptidase IV (DPP-IV) with 2.1% inhibition at 10^−5^ M [103]. 


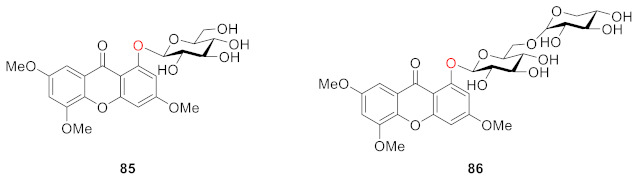


In 2013, Luo’s group isolated 7-hydroxy-3,4,8-trimethoxyxanthone-1-*O*-(*β*-d-glucoside) (**87**), 6-hydroxy-3,5-dimethoxyxanthone-1-*O*-(*β*-d-glucoside) (**88**), and 3,4,7,8-tetramethoxyxanthone-1-*O*-(*β*-d-glucoside) (**89**) from *Swertia mussotii*. These three compounds were found to have moderate anti-oxidant activity. Their oxygen radical absorbance capacity (ORAC) values at a concentration of 3.1 μM were 30.2 ± 0.2, 33.1 ± 0.2 and 33.2 ± 0.7, respectively. The experiment in this study also showed that the bio-activity of glycosylated xanthones was higher than that of xanthones without glycosylation [78].


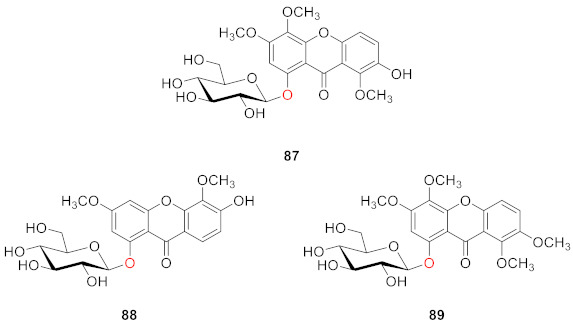


From an *n*-butanol fraction of *Swertia kouitchensis*, ten new xanthone glucosides, kouitchensides A–J (**90**–**99,** shown in Table 3), were isolated. The structures of these glucosides were determined by interpreting extensive spectroscopic data. In an in vitro test, compounds **91**, **93**, **94**, and **95** (IC_50_ values ranging from 126 to 451 μM) inhibited *α*-glucosidase activity more effectively than acarbose, the positive control (IC_50_ value of 627 μM) [104].


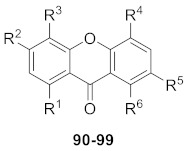


In 2014, seven new xanthone glucosides (**100**–**106,** shown in Table 4) were isolated from the *n*-butanol extract of *Swertia bimaculate*. Compounds **102**, **103**, and **106** were found to have significant *α*-glucosidase inhibitory activities in vitro (IC_50_ values of 142 µM, 136 µM, and 258 µM, respectively), and the assay showed that glucoside units at *C*-1 exhibited more potent inhibitory activity than the units located at *C*-8 [105].


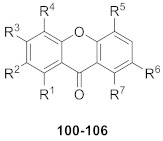


Hu’s group found a disaccharide substituted xanthone *O*-glucoside from the whole plant of *Lomatogonium carinthiacum* (Wulfen) Rchb. and identified its structure as 1,4,8-trimethoxyxanthone-6-*O*-*β*-d-glucoronyl-(1→6)-*O*-*β*-d-glucoside in 2014 (**107**) [106].


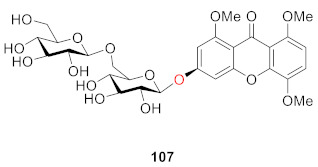


1,2-Dihydroxy-6-methoxyxanthone-8-*O*-*β*-d-xylopyranosyl (**108**) is one of the main constituents of petroleum ether and ethyl acetate extracts from *Swertia corymbosa* (*Gentinaceae*), a medicinal plant used in traditional Indian systems to treat diabetes. In STZ-induced diabetic rats, the compound aids in the management of diabetes and the prevention of its vascular complications and may be useful in the treatment of anti-hyperglycemia and anti-hyperlipidemia in diabetic patients [107].


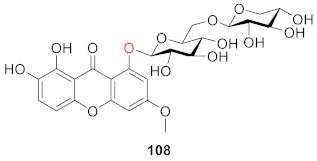


Six new tetrahydroxanthone glucosides (amarellins A–F (**109**–**114**)) were isolated from the aerial parts of the Mongolian medicinal plant *Gentianella amarelle* ssp. *acuta* (Gentianaceae) by Yoshiki Kashiwada and colleagues in 2016. Amarellins A–C (**109**–**111**) were assigned as 8-*O*-*β*-d-glucoside, 8-*O*-*β*-d-xyloside, and 1-*O*-*β*-d-glucoside of the *trans*-tetrahydroxanthone, respectively, while amarellins D–F (**112**–**114**) were elucidated to be 8-*O*-*β*-d-xyloside, 1-*O*-*β*-d-glucoside, and 3-*O*-*β*-d-glucoside of the *cis*-tetrahydroxanthone, respectively [108].


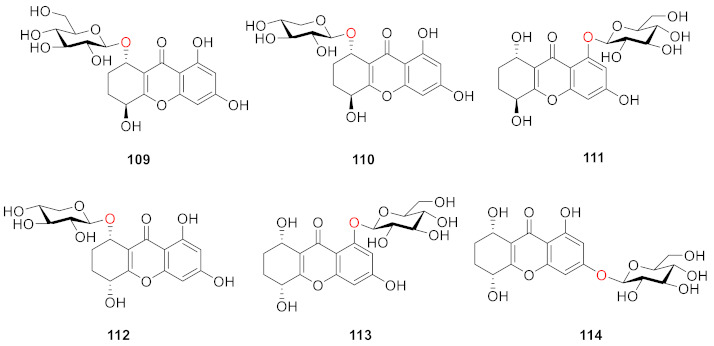


Kuang’s laboratory isolated a new compound, 5-hydroxy-3,4,6-trimethoxyxanthone-1-*O*-*β*-d-glucopyranoside (**115**), and two known compounds, norswertianolin (**72**) and swertianolin (**116**) [109,110,111,112] from *Gentianella acuta* (Michx.) Hulten in 2018 [46]. A mixture of extracts that included **72** and **116** was verified to provide protection against myocardial I/R injury through their anti-oxidative and anti-apoptotic effects [113].


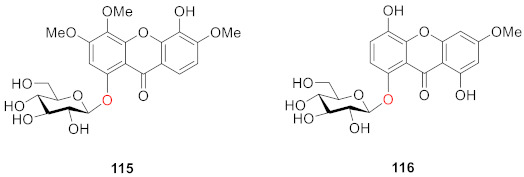


#### 2.2.2. Xanthone *O*-Glucoside from Clusiaceae

From the stem of *Poeciloneuron pauciflorum*, a new xanthone, 1,6-dihydroxy-7-methoxyxanthone 6-*O*-*β*-d-glucoside (**117**) was isolated by the Riswan group in 1997. The glucose moiety was located at *C*-6 of the xanthone [114].


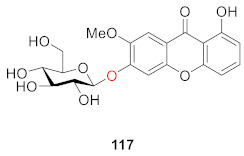


#### 2.2.3. Xanthone *O*-Glucoside from Hypericaceae

From the aerial part of *Hypericum japonicum*, one new xanthone glucoside, 1,5-dihydroxyxanthone-6-*O*-*β*-d-glucoside (**118**), was isolated from *Hypericum japonicum* in 1998 by Wu. Compound **118** was found to exert interesting coagulant activity in an in vitro test, showing prothrombin coagulation activity [18]. Zhang also isolated and identified 1,6-dihydroxyisojacereubin-5-*O*-*β*-d-glucoside (**119**), which is a tetracyclic compound containing a 2*H*-pyran ring, from the same plant in 2006 [115].


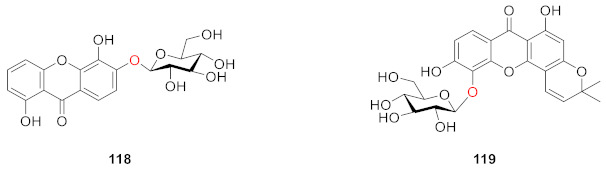


Ishiguro and colleagues isolated two new compounds, patuloside A (**120**) and patuloside B (**121**) in 1999 from cell suspension cultures of *Hypericum patulum* [116]. This is the first report on the isolation of 1,3,5,6-tetrahydroxyxanthone glucosides from cell suspension cultures of *H. patulum*.


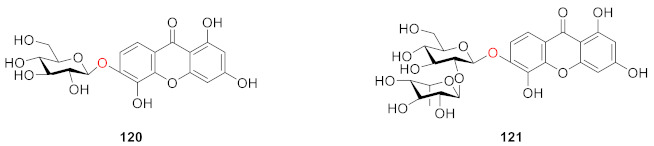


In 2000, Kitanov and Nedialkov extracted and identified an innovative compound from *Hypericum annulatum* and named it xanthohypericoside (**122**) [117].


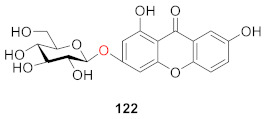


A phytochemical study on the aerial parts of *Hypericum elatoides* led to the isolation of five previously undescribed phenolic metabolites, hyperelatones E–H (**123**–**126**), along with tenuiside A (**127**) in 2019 by Gao’s group. Compound **123** has a hydroxyethyl group at the *C*-1 position and **126** is a compound with only a glucoside side chain. It was experimentally verified that **125**, **126** and **127** had neuroprotective activity and could improve the survival rate of P*C*-12 cells in a dose-dependent manner, among which **126** and **127** had the strongest activity. Compounds **125**, **126** and **127** also inhibited neuroinflammation induced by lipopolysaccharide (LPS) in BV-2 microglial cells without cytotoxicity to cells with IC_50_ values of 3.84 ± 0.15, 0.75 ± 0.02, and 1.39 ± 0.03 μM, respectively. In addition, **125**, **126**, and **127** showed stronger activity than **123** and **124** [118].


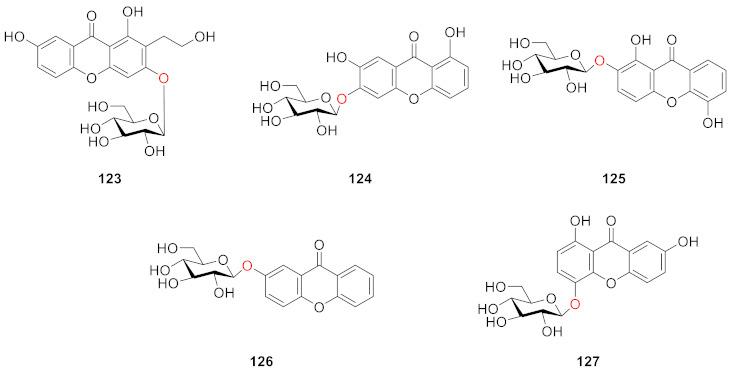


#### 2.2.4. Xanthone *O*-Glucoside from Iridaceae

An and coworkers separated 1-hydroxy-3,5-dimethoxy-xanthone-6-*O*-*β*-d-glucoside (**128**) from *Iris minutiaurea* Makino in 2016. To assess the anti-inflammatory activity of this compound, they measured its inhibitory rate of it on nitric oxide (NO) production, and tumor necrosis factor-α (TNF-*α*), interleukin-1*β* (IL-1*β*), and IL-6 release by LPS-induced RAW 264.7 macrophage cells. The results showed that the compound could exert an anti-inflammatory effect by inhibiting the production of the pro-inflammatory cytokine NO [119].


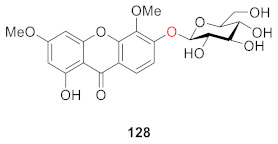


#### 2.2.5. Xanthone *O*-Glucoside from Polygalaceae

Li’s group isolated polycaudoside A (**129**) from the roots of *Polygala caudata* Reld et Wils in 1999. As seen from the structure, the glucoside side chains of **129** and **121** are the same, but the difference is that **121** has two more hydroxyl groups than **129** [120].


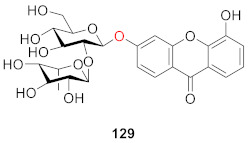


#### 2.2.6. Xanthone *O*-Glucoside from Polygonaceae 

In 2013, Nafady’s group isolated a new xanthone *O*-glucoside (**130**) from the methanol extract of the aerial part of the plant *Polygonum bellardii* growing in Egypt. The structure of the compound was 1,8-dihydroxy-3,6-dimethoxy-xanthone-5-*O*-[*α*-ʟ-rhamnopyranosyl-(1″→2′)]-*β*-d-glucopyranoside. The DPPH scavenging test of all obtained compounds found that **130** had certain anti-oxidant potential. The scavenging rates of **130** were 18.2 ± 1.56%, 28.4 ± 1.93%, 41.1 ± 0.99%, 51.0 ± 0.98%, and 66.1 ± 0.87% at concentrations of 10, 25, 50, 100, and 200 µg/mL, respectively, and the IC_50_ value was 79.3 ± 2.65 μM [121].


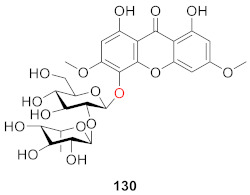


In 2005, Tu’ group isolated tricornosides B–F (**131**–**135**) from the roots of *Polygala tricornis.* With the exception of **134**, all of the compounds were diglucosides, and all of the remaining four compounds, with the exception of **131**, contained hydroxyl groups at the *C*-1 position [122].


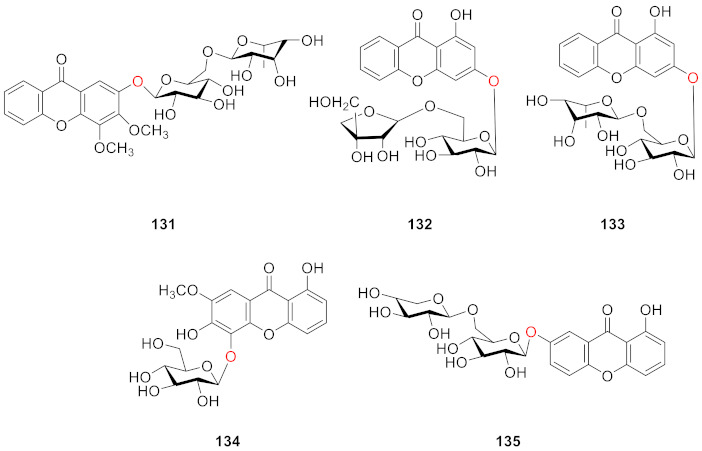


Polygalaxanthones IX (**136**) and X (**137**) were isolated from the cortexes of *Polygala tenuifolia* by Jiang’s group. Compound **136** was identified as 3-*O*-[*α*-ʟ-rhamnopyranosyl-(1→2)-*β*-d-glucopyranosyl]-1,7-dihydroxyxanthone and **137** was identified as 6-*O*-[*α*-ʟ-rhamnopyranosyl-(1→2)-*β*-d-glucopyranosyl]-1,2,3,7-tetramethoxyxanthone [67].


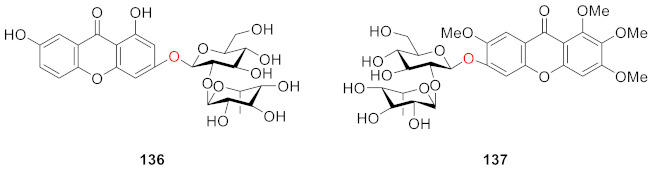


In 2008, two xanthone glucosides, polyhongkongenosides A (**138**) and B (**139**) and a known compound called polygalaxanthone V (**140**) [123], were isolated from *Polygala hongkongensis* [70].


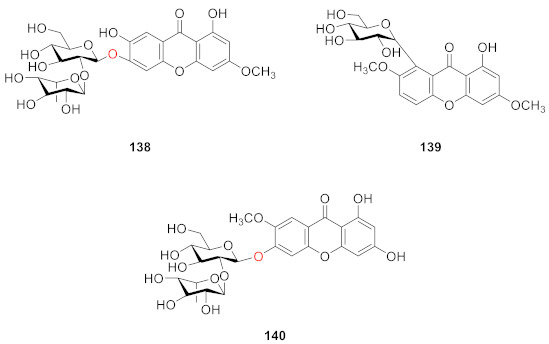


#### 2.2.7. Xanthone *O*-Glucoside from Polypodiaceae

3,5,7,8-Tetramethoxyxanthone-1-*O*-*β*-d-glucopyranoside (**141**) was isolated and identified from *Pyrrosia sheareri* (Bak.) Ching by Du and coworkers in 2019. Since *Pyrrosia* mainly contains xanthone *C*-glucoside, this compound can be used as a characteristic component of *Pyrrosia sheareri* to assist in the identification of *Pyrrosia sheareri* [124].


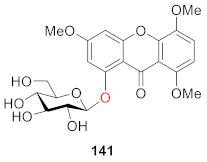


#### 2.2.8. Others

Xanthone glucosides have frequently been described in higher plants, but only a few reports that describe the presence of glucosides from lichens have been published. Rezanka and Dembitsky extracted and identified 16 compounds (**142**–**157**) from *Umbilicaria proboscidea* in 2003. As shown below, umbilicaxanthosides A (**142**) and B (**150**) are mono- and di-prenyl xanthones, and other compounds are their 6-*O*-acylated derivatives (**142**–**149, 150**–**157**) [125,126].


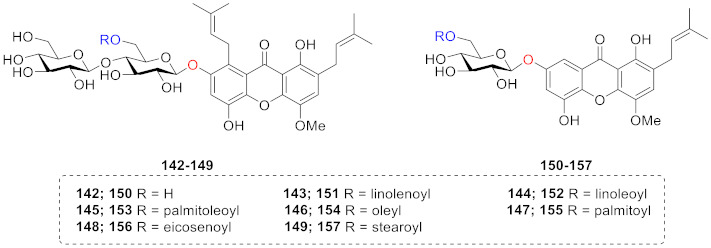


Microluside A (**158**) is a unique *O*-glycosylated disubstituted xanthone isolated from the broth culture of *Micrococcus sp.* EG45 cultivated from the Red Sea sponge *Spheciospongia vagabunda*. Anti-microbial activity evaluations showed that **158** exhibited anti-bacterial potential against *Enterococcus faecalis* JH212 and *Staphylococcus aureus* NCTC 8325 with MIC values of 10 and 13 μM, respectively [127].


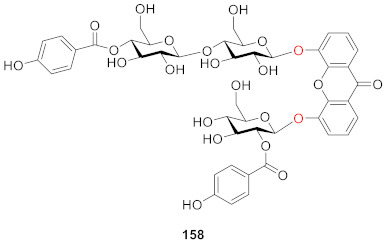


Recently, Xiong’s group isolated sporormielloside (**159**) from an EtOAc extract of *Sporormiella irregularis* in 2016. The presence of a methyl group in the structure of compound **159** is unusual [128].


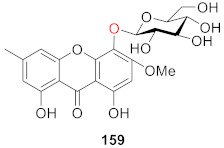


Recently, Yoneyama’s team isolated and identified a new compound (**160**) (whose structure is 3-*O*-(4-*O*-methyl-*β*-d-glucopyranosyl) xanthone) from the culture of Conoideocrella luteorostrata NBRC106950 in 2021 [129]. To date, only two xanthone glucosides with methyl substituents have been isolated, including **159**.


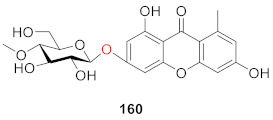


## 3. NMR Difference of Xanthone Glucosides

After investigation on the NMR data of xanthone *C*-glucosides and xanthone *O*-glucosides reported in the literature, it was discovered that there was no significant difference in the chemical shift of protons in ^1^H NMR spectrum. However, the ^13^C NMR data showed regular difference in the chemical shifts of *C*-1 of sugars which connected to the xanthone structures.

Generally, the chemical shifts of the sugar group appear among the range of δ 60–110 (^13^C NMR). It was found that the chemical shift of *C*-1 on the sugar group in xanthone *C*-glucosides is obviously smaller than that of xanthone *O*-glucosides. The chemical shift value of the former is basically distributed around δ 74, while that of the latter is mainly distributed between δ 100–110. Conversely, for the chemical shifts of *C*-3 and *C*-5 of sugar group, xanthone *C*-glucosides is slightly greater than xanthone *O*-glucosides. For example, neomangiferin is a compound bearing both *C*- and *O*-glycosides. The chemical shifts of *C*-1, *C*-3, and *C*-5 of the sugar group via *O*-linker are 103.1, 76.5, and 77.2, respectively, while the chemical shifts of *C*-1, *C*-3, and *C*-5 via *C*-linker are 73.2, 79.1, and 81.4, respectively [31]. For more examples, please see the chemical shifts listed in the Table 5 below.

## 4. Synthesis of Xanthone Glucosides or Derivatives

**Scheme 1:**
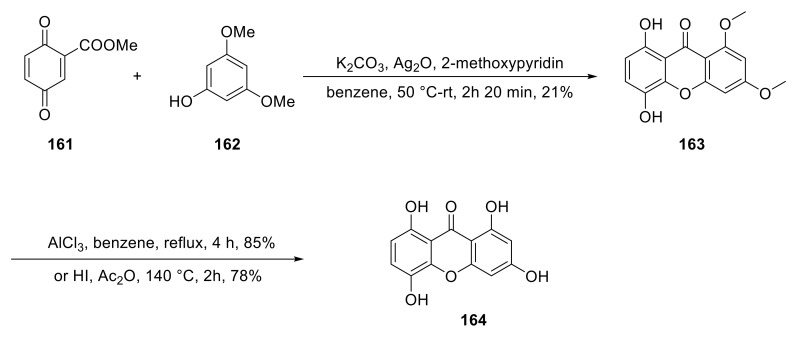

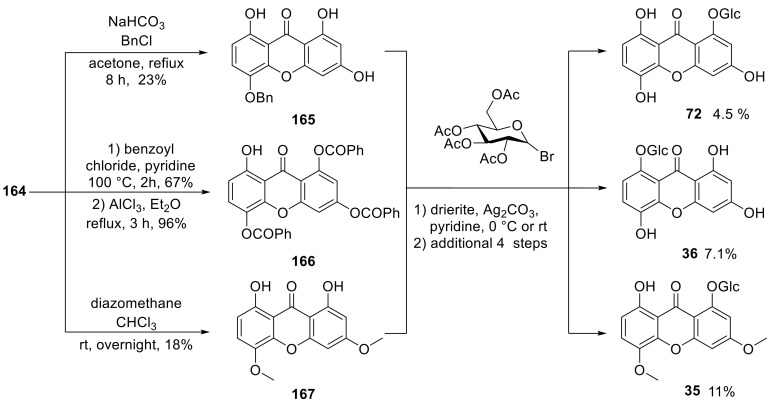
The synthesis of **35**, **36** and **72**.

The first synthesis of xanthone glucosides was accomplished by Wagner in 1985 (Scheme 1) [130]. Three xanthones, 1-(*β*-d-glucosyloxy)-8-hydroxy-3,5-dimethoxyxanthone (**35**) [80], 8-(*β*-d-glucosyloxy)-1,3,5-trihydroxyxanthone (**36**) [81] and norswertianolin (**72**) [92], were synthesized in one sequence. Starting with the nucleophilic addition of 3,5-dimethoxyphenol (**162**) to 2-methoxycarbonyl-1,4-benzoquinone (**161**), 5,8-dihydroxy-1,3-dimethoxyxanthone (**163**) was obtained at 21% yield. The compound 1,3,5,8-tetrahydroxyxanthone (**164**) was completed by refluxing **163** with AlCl_3_ in benzene for 4 h or heating **163** with HI and Ac_2_O at 140 °C Compound **164** then went through different steps to form three compounds: 5-benzyloxy-l,3,8-trihydroxyxanthone (**165**), 1,3,5-tribenzoyloxy-8-hydroxyxanthone (**166**), and 1,8-dihydroxy-3,5-dimethoxy (**167**). Then, taking **165**–**167** as aglycons, coupling with *α*-acetobromoglucose successfully afforded the corresponding products **72**, **36**, and **35**. 

**Scheme 2:**
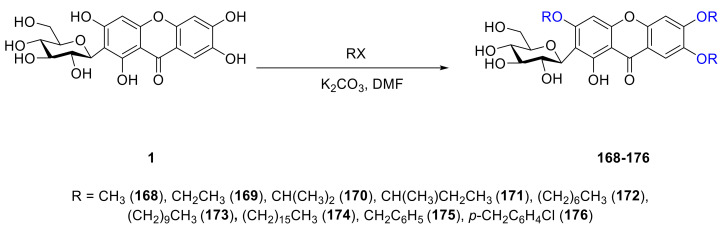
The synthesis of mangiferin derivatives.

The structure of mangiferin (**1**) is 2-(*β*-d-glucosyloxy)-1,3,6,7-tetrahydroxyxanthone, which is distributed in a variety of plants and has demonstrated many biological activities. To improve the solubility of **1**, several mangiferin derivatives were synthesized by Wu and coworkers (Scheme 2). They used nucleophilic substitution to add alkyl or benzyl groups to the skeleton of mangiferin and nine derivatives **168**–**176** were obtained [131].

**Scheme 3:**
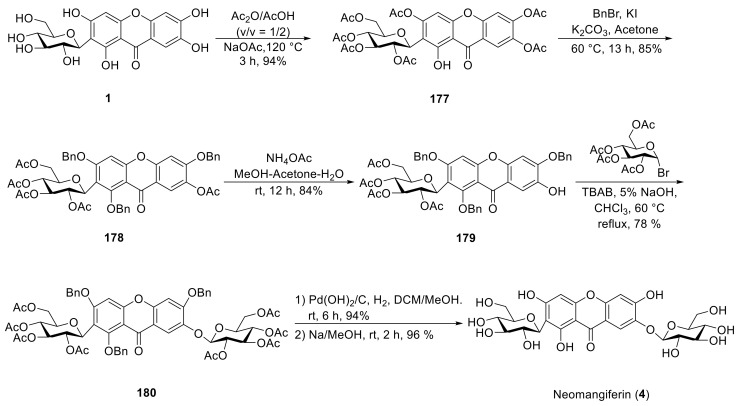
The synthesis of neomangiferin.

Neomangiferin (**4**) is a derivative of **1**. Li and coworkers solved the problem of hydroxyl selectivity and realized the semi-synthesis of **4** from **1** in 2014 (Scheme 3). First, compound **177** was synthesized by acylation in high yield, which is a suitable intermediate for selective benzylation at the 1-, 3- and 6-positions. After de-acylation, only the remaining 7-OH can be coupled with *α*-d-glucopyranosyl bromide under optimized conditions to give the corresponding product **4** after the removal of all the protective groups [132].

**Scheme 4:**
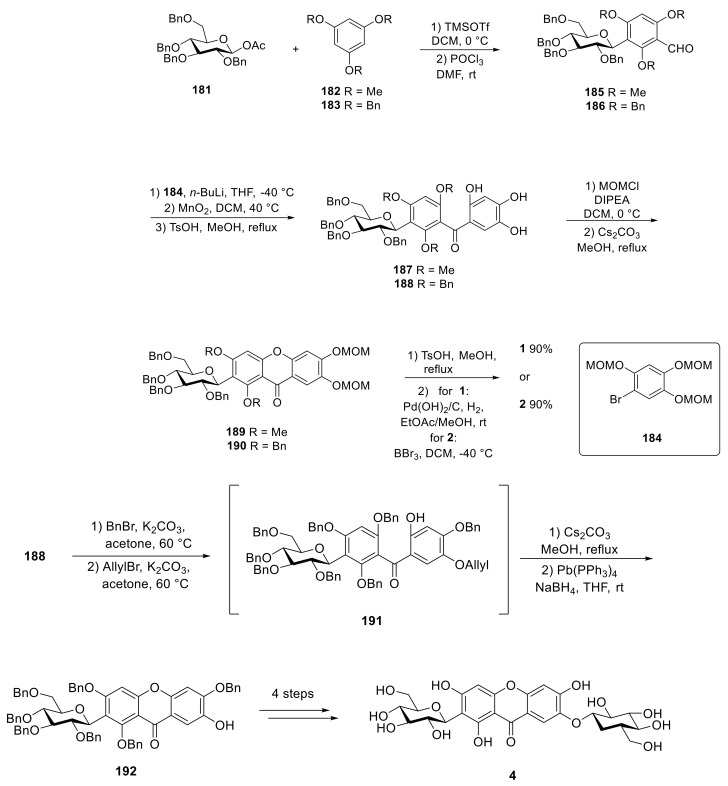
The synthesis of mangiferin, homomangiferin, and neomangiferin.

As a continuing work, the Li group completed the total synthesis of three xanthone glucosides including **1**, homomangiferin (**2**) and **4** using an alternative method in 2016. They chose tetrabenzylglucose (**181**), phloroglucinol derivatives (**182**–**183**) and bromobenzene derivatives (**184**) as the starting materials. Compounds **1** and **2** were synthesized by a series of steps, including glycosylation, Vilsmeier formylation, de-protection, selective reprotection, and ring formation reactions. Then, according to the research in 2014, the construction of **4** was completed (Scheme 4) [133].

In addition to chemical methods, enzyme catalysis can also be used to synthesize xanthone glucosides. For example, Zarena et al. used enzyme catalysis to achieve glycosylation of α−mangostin (**193**) in a supercritical carbon dioxide system [134], and Sohng completed the diversified glycosylation of **193** by a one-pot enzymatic catalysis [135]. In addition, Kim and coworkers modified **1** with glucansucrase to obtain the disglycation product mangiferin-(1→6)-α-d-glucopyranoside (**194**), thus improving the activity and solubility of mangiferin [24].


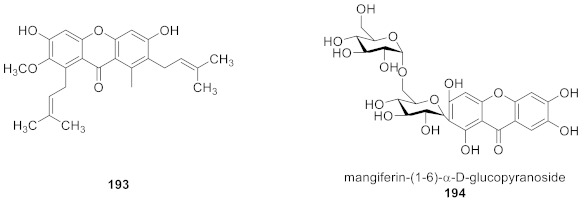


## 5. Conclusions and Outlook

In this review, we summarized 160 xanthone glucosides, of which xanthone *O*-glucoside was the most abundant (136 included). These compounds are derived from a variety of sources, with mangiferin being the most widely distributed and having the most investigated pharmacological activities. There was no significant difference in bioactivity between glucosylxanones and xanthones, but glycosylation can usually improve bioactivity.

We reviewed 93 monosaccharide xanthone glucosides and 66 disaccharide xanthone glucosides. Disaccharide xanthone glucosides are composed primarily of two glucose or glucose and xylose sugars, with a small amount of glucose combined with rhamnose, apiose, or arabinose. In terms of sugar binding sites, xanthone *C*-glucosides have glucosyl groups primarily at *C*-2, whereas xanthone *O*-glucosides have glucosyl groups primarily at *C*-1. Hydroxyl and methoxy groups are the most common substituents on the xanthone skeleton. Only two compounds out of 160 contain a methyl group (**159** and **160**). Prenylated xanthone glucosides are also extremely rare and have only been discovered in lichens (**142**–**157**). With the exception of a few examples containing tetrahydroxanthones, xanthone glucosides all have a xanthone skeleton (**80**–**81, 109**–**114**).

Despite the fact that a number of xanthone glucosides have been discovered, the medicinal study and health benefits of this type of compound have largely been limited to mangiferin. Synthesis and structural modification based on xanthones and glucosyl groups are also underdeveloped. Future research could concentrate on the synthesis of xanthone glucoside derivatives and the investigation of their pharmacological activities.

## Figures and Tables

**Table 1:** The structure of compounds **10**–**15**.

	R^1^	R^2^
**10**	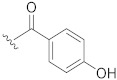	H
**11**	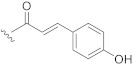	H
**12**	H	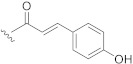
**13**	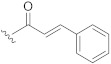	H
**14**	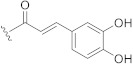	H
**15**	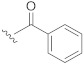	H

**Table 2:** The structure of compounds **56**–**63**.

	R^1^	R^2^	R^3^	R^4^	R^5^	R^6^
**56**	H	OMe	Gentiobiosyl	OMe	OMe	H
**57**	H	OMe	Primeverosyl	OMe	OMe	H
**58**	OMe	H	Gentiobiosyl	OMe	OMe	OMe
**59**	H	OMe	Primeverosyl	OMe	OMe	OMe
**60**	OMe	OH	Gentiobiosyl	OMe	OMe	OMe
**61**	OMe	OH	Primeverosyl	OMe	OMe	OMe
**62**	OMe	OMe	Gentiobiosyl	OMe	OMe	OMe
**63**	OMe	OMe	Primeverosyl	OMe	OMe	OMe

**Table 3:** The structure of compounds **90**–**99**.

	R^1^	R^2^	R^3^	R^4^	R^5^	R^6^
**90**	*O*-glc(6–1)-xyl	OMe	OMe	H	OH	OMe
**91**	*O*-glc(6–1)-xyl	OMe	OMe	H	OMe	OMe
**92**	*O*-glc	OMe	OH	H	OH	OMe
**93**	*O*-glc(6–1)-xyl	OMe	H	OMe	H	OMe
**94**	*O*-glc(6–1)-xyl	OMe	H	OMe	OMe	OMe
**95**	OH	*O*-glc(6–1)-xyl	OMe	OMe	H	OH
**96**	*O*-glc(6–1)-glc	OMe	H	OMe	H	H
**97**	*O*-glc(6–1)-glc	OMe	H	OMe	H	OH
**98**	OH	OMe	H	H	*O*-glc(2–1)-rha	OH
**99**	OH	OMe	H	H	*O*-rha	*O*-glc

**Table 4:** The structure of compounds **100**–**106**.

	R^1^	R^2^	R^3^	R^4^	R^5^	R^6^	R^7^
**100**	*O*-glc(6–1)-xyl	OMe	OMe	OMe	OMe	H	OH
**101**	OH	OMe	OMe	OMe	OMe	H	*O*-glc(6–1)-xyl
**102**	*O*-glc(6–1)-xyl	H	OMe	OMe	OMe	H	OH
**103**	OH	H	OMe	OMe	OMe	H	*O*-glc(6–1)-xyl
**104**	*O*-glc(6–1)-xyl	H	OH	OMe	OMe	H	OH
**105**	*O*-glc(6–1)-glc	H	OH	OMe	OMe	H	OH
**106**	*O*-glc(6–1)-glc	H	OH	OMe	OMe	H	OH

**Table 5:** ^13^C NMR data of typical compounds.

Category	Compound [Ref.]	*C*-1′	*C*-3′	*C*-5′
Xanthone *C*-glucoside	**1** [34]	73.6	79.5	82.1
	**10** [62]	75.4	81.6	82.7
	**22** [69]	74.04	79.60	80.55
	**29** [77]	74.1	77.9	81.3
	**31** [78]	74.4	80.3	82.9
Xanthone *O*-glucoside	**69** [92]	102	76.4	76.3
	**79** [77]	103.6	76.0	77.5
	**87** [78]	103.7	76.2	77.8
	**119** [115]	105.4	77.2	76.0
	**123** [118]	100.4	76.4	77.2

## References

[1] Fiesel T., Gaid M., Muller A., Bartels J., El-Awaad I., Beuerle T., Ernst L., Behrends S., Beerhues L. (2015). Molecular Cloning and Characterization of a Xanthone Prenyltransferase from Hypericum calycinum Cell Cultures. Molecules.

[2] Mandal S., Das P.C., Joshi P.C. (1992). Naturally-occurring xanthones from terrestrial flora. J. Indian Chem. Soc..

[3] Peres V., Nagem T.J. (1997). Naturally occurring, pentaoxygenated, hexaoxygenated and dimeric xanthones: A literature survey. Quim. Nova.

[4] Nhan N.T., Nguyen P.H., Tran M.H., Nguyen P.D., Tran D.T., To D.C. (2021). Anti-inflammatory xanthone derivatives from Garcinia delpyana. J. Asian Nat. Prod. Res..

[5] Banik K., Harsha C., Bordoloi D., Lalduhsaki Sailo B., Sethi G., Leong H.C., Arfuso F., Mishra S., Wang L., Kumar A.P. (2018). Therapeutic potential of gambogic acid, a caged xanthone, to target cancer. Cancer Lett..

[6] Moon K.M., Kim C.Y., Ma J.Y., Lee B. (2019). Xanthone-related compounds as an anti-browning and antioxidant food additive. Food Chem..

[7] Akao Y., Nakagawa Y., Iinuma M., Nozawa Y. (2008). Anti-Cancer Effects of Xanthones from Pericarps of Mangosteen. Int. J. Mol. Sci..

[8] Rukachaisirikul V., Kamkaew M., Sukavisit D., Phongpaichit S., Sawangchote P., Taylor W.C. (2003). Antibacterial xanthones from the leaves of Garcinia nigrolineata. J. Nat. Prod..

[9] He L., Zhu C.F., Yuan Y., Xu Z.F., Qiu S.X. (2014). Specific glycosylated metabolites of α-mangostin by Cunninghamella blakesleana. Phytochem. Lett..

[10] Gales L., Damas A.M. (2005). Xanthones–A Structural Perspective. Curr. Med. Chem..

[11] Klein L.C., Campos A., Niero R., Correa R., Vander Heyden Y., Cechinel V. (2020). Xanthones and Cancer: From Natural Sources to Mechanisms of Action. Chem. Biodivers..

[12] Shan T., Ma Q., Guo K., Liu J., Li W., Wang F., Wu E. (2011). Xanthones from Mangosteen Extracts as Natural Chemopreventive Agents: Potential Anticancer Drugs. Curr. Mol. Med..

[13] Han Q.B., Xu H.X. (2009). Caged Garcinia Xanthones: Development since 1937. Curr. Med. Chem..

[14] El-Seedi H.R., El-Ghorab D.M.H., El-Barbary M.A., Zayed M.F., Goransson U., Larsson S., Verpoorte R. (2009). Naturally Occurring Xanthones; Latest Investigations: Isolation, Structure Elucidation and Chemosystematic Significance. Curr. Med. Chem..

[15] Na Y. (2009). Recent cancer drug development with xanthone structures. J. Pharm. Pharmacol..

[16] Araujo J., Fernandes C., Pinto M., Tiritan M.E. (2019). Chiral Derivatives of Xanthones with Antimicrobial Activity. Molecules.

[17] Vieira L.M.M., Kijjoa A. (2005). Naturally-Occurring Xanthones: Recent Developments. Curr. Med. Chem..

[18] Wu Q.L., Wang S.P., Du L.J., Yang J.S., Xiao P.G. (1998). Xanthones from Hypericum japonicum and H-Henryi. Phytochemistry.

[19] Perest V., Nagem T.J. (1997). Trioxygenated naturally occurring xanthones. Phywchemistry.

[20] Valdir P., Nagem T.J., de Oliveira F.F. (2000). Tetraoxygenated naturally occurring xanthones. Phytochemistry.

[21] Mangangcha I.R., Singh R.K.B., Lebeche D., Ali S. (2021). Xanthone glucoside 2-beta-d-glucopyranosyl-1,3,6,7-tetrahydroxy-9*H*-xanthen-9-one binds to the ATP-binding pocket of glycogen synthase kinase 3 beta and inhibits its activity: Implications in prostate cancer and associated cardiovascular disease risk. J. Biomol. Struct. Dyn..

[22] Ghosal S., Sharma P.V., Chaudhuri R.K. (1974). Chemical constituents of gentianaceae. X. Xanthone-*O*-glucosides of *Swertia purpurascens* Wall. J. Pharm. Sci..

[23] Kren V., Martinkova L. (2001). Glycosides in medicine: “The role of glycosidic residue in biological activity”. Curr. Med. Chem..

[24] Septiana I., Nguyen T.T.H., Lim S., Lee S., Park B., Kwak S., Park S., Kim S.B., Kim D. (2020). Enzymatic synthesis and biological characterization of a novel mangiferin glucoside. Enzym. Microb. Technol..

[25] Feng S.T., Wang Z.Z., Yuan Y.H., Sun H.M., Chen N.H., Zhang Y. (2019). Mangiferin: A multipotent natural product preventing neurodegeneration in Alzheimer’s and Parkinson’s disease models. Pharmacol. Res..

[26] Jo C., Yoon K.Y., Jang E.J., Kim T.H. (2016). Degradation products of mangiferin by gamma irradiation with inhibitory effects on NO production. Biosci. Biotechnol. Biochem..

[27] Yoshimi N., Matsunaga K., Katayama M., Yamada Y., Kuno T., Qiao Z., Hara A., Yamahara J., Mori H. (2001). The inhibitory effects of mangiferin, a naturally occurring glucosylxanthone, in bowel carcinogenesis of male F344 rats. Cancer Lett..

[28] Kim G.E., Kang H.K., Seo E.S., Jung S.H., Park J.S., Kim D.H., Kim D.W., Ahn S.A., Sunwoo C., Kim D. (2012). Glucosylation of the flavonoid, astragalin by Leuconostoc mesenteroides B-512FMCM dextransucrase acceptor reactions and characterization of the products. Enzym. Microb. Technol..

[29] Faizi S., Zikr-ur-Rehman S., Naz A., Versiani M.A., Dar A., Naqvi S. (2012). Bioassay-guided studies on Bombax ceiba leaf extract: Isolation of shamimoside, a new antioxidant xanthone *C*-glucoside. Chem. Nat. Compd..

[30] Finnegan R.A., Stephani R.A., Ganguli G., Ganguly S.N., Bhattacharya A.K. (1968). Occurrence of mangiferin in Hiptage madablota Geartn. J. Pharm. Sci..

[31] Hong Y.F., Han G.Y., Guo X.M. (1997). Isolation and structure determination of xanthone glycosides of *Anemarrhena asphodeloides*. Acta Pharm. Sin..

[32] Iwashina T., Kitajima J., Shiuchi T., Itou Y. (2005). Chalcones and other flavonoids from *Asarum* sensu lato (Aristolochiaceae). Biochem. Syst. Ecol..

[33] Zhang Y.B., Xu X.J., Liu H.M. (2006). Chemical constituents from *Mahkota dewa*. J. Asian Nat. Prod. Res..

[34] Talamond P., Mondolot L., Gargadennec A., de Kochko A., Hamon S., Fruchier A., Campa C. (2008). First report on mangiferin (*C*-glucosyl-xanthone) isolated from leaves of a wild coffee plant, *Coffea pseudozanguebariae* (Rubiaceae). Acta Bot. Gallica.

[35] Li L., Li M.H., Zhang N., Huang L.Q. (2011). Chemical constituents from *Lomatogonium carinthiacum* (Gentianaceae). Biochem. Syst. Ecol..

[36] Khare P., Shanker K. (2016). Mangiferin: A review of sources and interventions for biological activities. Biofactors.

[37] Wei Z., Yan L., Chen Y., Bao C., Deng J., Deng J. (2016). Mangiferin inhibits macrophage classical activation via downregulating interferon regulatory factor 5 expression. Mol. Med. Rep..

[38] Feng Z.L., Lu X.Q., Gan L.S., Zhang Q.W., Lin L.G. (2020). Xanthones, A Promising Anti-Inflammatory Scaffold: Structure, Activity, and Drug Likeness Analysis. Molecules.

[39] Sha H., Zeng H.R., Zhao J., Jin H.Y. (2019). Mangiferin ameliorates gestational diabetes mellitus-induced placental oxidative stress, inflammation and endoplasmic reticulum stress and improves fetal outcomes in mice. Eur. J. Pharmacol..

[40] Dar A., Faizi S., Naqvi S., Roome T., Zikr-ur-Rehman S., Ali M., Firdous S., Moin S.T. (2005). Analgesic and antioxidant activity of mangiferin and its derivatives: The structure activity relationship. Biol. Pharm. Bull..

[41] He L.Y., Peng X.F., Zhu J.F., Chen X., Liu H., Tang C.Y., Dong Z., Liu F.Y., Peng Y.M. (2014). Mangiferin Attenuate Sepsis-Induced Acute Kidney Injury via Antioxidant and Anti-Inflammatory Effects. Am. J. Nephrol..

[42] PardO-Andreu G.L., Barrios M.F., Curti C., Hernandez I., Merino N., Lemus Y., Martinez L., Riano A., Delgado R. (2008). Protective effects of *Mangifera indica* L. extract (Vimang), and its major component mangiferin, on iron-induced oxidative damage to rat serum and liver. Pharmacol. Res..

[43] Zheng H.H., Luo C.T., Chen H.R., Lin J.N., Ye C.L., Mao S.S., Li Y.L. (2014). Xanthones from *Swertia mussotii* as Multitarget-Directed Antidiabetic Agents. Chemmedchem.

[44] Muruganandan S., Srinivasan K., Gupta S., Gupta P.K., Lal J. (2005). Effect of mangiferin on hyperglycemia and atherogenicity in streptozotocin diabetic rats. J. Ethnopharmacol..

[45] Miura T., Ichiki H., Hashimoto I., Iwamoto N., Kato M., Kubo M., Ishihara E., Komatsu Y., Okada M., Ishida T. (2001). Antidiabetic activity of a xanthone compound, mangiferin. Phytomedicine.

[46] Wang Z.B., Wu G.S., Yu Y., Liu H., Yang B.Y., Kuang H.X., Wang Q.H. (2018). Xanthones isolated from *Gentianella acuta* and their protective effects against H2O2-induced myocardial cell injury. Nat. Prod. Res..

[47] Muruganandan S., Gupta S., Kataria M., Lal J., Gupta P.K. (2002). Mangiferin protects the streptozotocin-induced oxidative damage to cardiac and renal tissues in rats. Toxicology.

[48] Prabhu S., Jainu M., Sabitha K.E., Devi C.S.S. (2006). Effect of mangiferin on mitochondrial energy production in experimentally induced myocardial infarcted rats. Vasc. Pharmacol..

[49] Zeng Z., Lin C.J., Wang S.W., Wang P.F., Xu W.W., Ma W.Q., Wang J.L., Xiang Q., Liu Y.T., Yang J.M. (2020). Suppressive activities of mangiferin on human epithelial ovarian cancer. Phytomedicine.

[50] Deng Q., Tian Y.X., Liang J.J. (2018). Mangiferin inhibits cell migration and invasion through Rac1/WAVE2 signalling in breast cancer. Cytotechnology.

[51] Lv J.Z., Wang Z.J., Zhang L., Wang H.L., Liu Y.D., Li C.Y., Deng J.G., Yi W., Bao J.K. (2013). Mangiferin Induces Apoptosis and Cell Cycle Arrest in MCF-7 Cells Both in vitro and in vivo. J. Anim. Vet. Adv..

[52] Pinto M.M.M., Sousa M.E., Nascimento M.S.J. (2005). Xanthone derivatives: New insights in biological activities. Curr. Med. Chem..

[53] Sato T., Kawamoto A., Tamura A., Tatsumi Y., Fujii T. (1992). Mechanism of antioxidant action of pueraria glycoside (PG)-1 (an isoflavonoid) and mangiferin (a xanthonoid). Chem. Pharm. Bull..

[54] Vyas A., Syeda K., Ahmad A., Padhye S., Sarkar F.H. (2012). Perspectives on Medicinal Properties of Mangiferin. Mini-Rev. Med. Chem..

[55] Andreu G.P., Delgado R., Velho J.A., Curti C., Vercesi A.E. (2005). Iron complexing activity of mangiferin, a naturally occurring glucosylxanthone, inhibits mitochondrial lipid peroxidation induced by Fe^2+^-citrate. Eur. J. Pharmacol..

[56] Aritomi M., Kawasaki T. (1970). A new xanthone *C*-glucoside, position isomer of mangiferin, from *Anemarrhena asphodeloides* Bunge. Chem. Pharm. Bull..

[57] Kokotkiewicz A., Luczkiewicz M., Pawlowska J., Luczkiewicz P., Sowinski P., Witkowski J., Bryl E., Bucinski A. (2013). Isolation of xanthone and benzophenone derivatives from *Cyclopia genistoides* (L.) Vent. (honeybush) and their pro-apoptotic activity on synoviocytes from patients with rheumatoid arthritis. Fitoterapia.

[58] Lim S.M., Kang G.D., Jeong J.J., Choi H.S., Kim D.H. (2016). Neomangiferin modulates the Th17/Treg balance and ameliorates colitis in mice. Phytomedicine.

[59] Arisawa M., Morita N., Kondo Y., Takemoto T. (1973). The constiuents of *Iris florentina* L. (3). structure of irisxanthone, a know *C*-glucosylxanthone. Chem. Pharm. Bull..

[60] Abdel-Mageed W.M., Al-Wahaibi L.H., Al-Saleem M.S.M., Gouda Y.G., Abdel-Kader M.S., Ibraheim Z.Z. (2018). Phytochemical and chemotaxonomic study on *Iris albicans* Lange leaves. Biochem. Syst. Ecol..

[61] Bukvicki D., Novakovic M., Ab Ghani N., Marin P.D., Asakawa Y. (2018). Secondary metabolites from endemic species *Iris adriatica* Trinajstic ex Mitic (Iridaceae). Nat. Prod. Res..

[62] Xie G.Y., Chen Y.J., Wen R., Xu J.Y., Wu S.S., Qin M.J. (2014). Chemical constituents from rhizomes of *Iris germanica*. China J. Chin. Mater. Med..

[63] Alkhalil S., Tosa H., Iinuma M. (1995). A xanthone *C*-glycoside from *Iris Nigricans*. Phytochemistry.

[64] Pauletti P.M., Castro-Gamboa I., Silva D.H.S., Young M.C.M., Tomazela D.M., Eberlin M.N., Bolzani V.D. (2003). New antioxidant *C*-glucosylxanthones from the stems of *Arrabidaea samydoides*. J. Nat. Prod..

[65] Martin F., Hay A.E., Cressend D., Reist M., Vivas L., Gupta M.P., Carrupt P.A., Hostettmann K. (2008). Antioxidant *C*-Glucosylxanthones from the Leaves of *Arrabidaea patellifera*. J. Nat. Prod..

[66] Miyase T., Noguchi H., Chen X.M. (1999). Sucrose Esters and Xanthone *C*-Glycosides from the Roots of *Polygala sibirica*. J. Nat. Prod..

[67] Jiang Y., Zhang W., Tu P.F., Xu X.J. (2005). Xanthone Glycosides from *Polygala tenuifolia* and Their Conformational Analyses. J. Nat. Prod..

[68] Chang H.T., Tu P.F. (2007). New oligosaccharide esters and xanthone *C*-glucosides from *Polygala telephioides*. Helv. Chim. Acta.

[69] Tsujimoto T., Nishihara M., Osumi Y., Hakamatsuka T., Goda Y., Uchiyama N., Ozeki Y. (2019). Structural Analysis of Polygalaxanthones, *C*-Glucosyl Xanthones of *Polygala tenuifolia* Roots. Chem. Pharm. Bull..

[70] Wu J.F., Chen S.B., Gao J.C., Song H.L., Wu L.J., Chen S.L., Tu P.F. (2008). Xanthone glycosides from herbs of *Polygala hongkongensis* Hemsl and their antioxidant activities. J. Asian Nat. Prod. Res..

[71] Jankovic T., KrstiC-Milosevic D., Aljancic I., Savikin K., Menkovic N., Radanovic D., Milosavljevic S. (2009). Phytochemical re-investigation of *Gentiana utriculosa*. Nat. Prod. Res..

[72] Schaufelberger D., Hostettmann K. (1988). Chemistry and pharmacology of *Gentiana lactea*. Planta Med..

[73] Fujita M., Inoue T. (1982). Studies on the Constituents of *Iris florentina* L. II. *C*-Glucosides of Xanthones and Flavones from the Leaves. Chem. Pharm. Bull..

[74] Shi T.X., Wang S., Zeng K.W., Tu P.F., Jiang Y. (2013). Inhibitory constituents from the aerial parts of *Polygala tenuifolia* on LPS-induced NO production in BV2 microglia cells. Bioorg. Med. Chem. Lett..

[75] Tan P., Hou C.Y., Liu Y.L., Lin L.J., Cordell G.A. (1991). Swertipunicoside. The First Bisxanthone *C*-Glycoside. J. Org. Chem..

[76] Tan P., Hou C.Y., Liu Y.L., Lin L.J., Cordell G.A. (1992). 3-*O*-demethylswertipunicoside from *Swertia punicea*. Phytochemistry.

[77] Du X.G., Wang W., Zhang S.P., Pu X.P., Zhang Q.Y., Ye M., Zhao Y.Y., Wang B.R., Khan I.A., Guo D.A. (2010). Neuroprotective Xanthone Glycosides from *Swertia punicea*. J. Nat. Prod..

[78] Luo C.T., Mao S.S., Liu F.L., Yang M.X., Chen H., Kurihara H., Li Y. (2013). Antioxidant xanthones from *Swertia mussotii*, a high altitude plant. Fitoterapia.

[79] Zhang L., Zou D.Z., Bai S., Li Z.H., Zhang C.H., Li M.H. (2016). Chemical constituents from *Gentianella turkestanorum* (Gentianaceae). Biochem. Syst. Ecol..

[80] Abdel-Mageed W.M., Bayoumi S.A.H., Chen C., Vavricka C.J., Li L., Malik A., Dai H., Song F., Wang L., Zhang J. (2014). Benzophenone *C*-glucosides and gallotannins from mango tree stem bark with broad-spectrum anti-viral activity. Bioorg. Med. Chem..

[81] Stout G.H., Balkenhol W.J. (1969). Xanthones of the Gentianaceae-I *Frasera caroliniensis* Walt. Tetrahedron.

[82] Tomimori T., Komatsu M. (1969). Studies on the Constituents of *Swertia japonica*. VI. on the Flavonoid and Xanthone Constituents of *Swertia randaiensis*. HAYATA amd S. swertopsis MAKINO. Yakugaku Zasshi.

[83] Tomimori T., Yoshizaki M., Nanba T. (1973). Studies on the Nepalese Crude Drugs.I. On the Flavonoid and Xanthone Constituents of the Plants of *Swertia* spp.. Yakugaku Zasshi.

[84] Hostettmann K., Tabacchi R., Jacot-Guillarmod A. (1974). Contribution à la phytochimie du genre *Gentiana*, VI. Etude des xanthones dans les feuilles de *Gentiana bavarica* L.. Helv. Chim. Acta.

[85] Hostettmann K., Miura I. (1977). A New Xanthone Diglucoside from *Swertia perennis* L.. Helv. Chim. Acta.

[86] Ghosal S., Sharma P.V., Jaiswal D.K. (1978). Chemical constituents of Gentianaceae XXIII: Tetraoxygenated and pentaoxygenated xanthones and xanthone *O*-glucosides of *Swertia angustifolia* Buch.-Ham. J. Pharm. Sci..

[87] Dhasmana H., Garg H.S. (1989). Two xanthone glucosides from *Halenia elliptica*. Phytochemrstry.

[88] Sun H.F., Hu B.L., Ding J.Y., Fan S.F. (1991). The glucosides from *Swertia mussotii* Franch. Acta Bot. Sin..

[89] Recio-Iglesias M.C., Marston A., Hosteyitman K. (1992). Xanthones and secoiridoid glucosides of *Halenia campanulata*. Phytochemistry.

[90] Rodriguez S., Wolfender J.L., Odontuya G., Purev O., Hostettmann K. (1995). Xanthones, secoiridoids and flavonoids from *Halenia corniculata*. Phytochemistry.

[91] Otsuka H., Triptexanthosides A.-E. (1999). Xanthone Glycosides from Aerial Parts of *Tripterospermum japonicum*. Chem. Pharm. Bull..

[92] Menkovic N., Savikin-Fodulovic K., Bulatovic V., Aljancic I., Juranic N., Macura S., Vajs V., Milosavljevic S. (2002). Xanthones from *Swertia punctata*. Phytochemistry.

[93] Zeng G.Y., Tan G.S., Xu K.P., Xu X.P., Li F.S., Tan J.B., Hu G.Y. (2004). Water-soluble chemical constituents of *Swertia davidi* Franch. Acta Pharm. Sin..

[94] Kaldas M., Hostettmann K., Jacot-Guillarmod A. (1974). Contribution à la phytochimie du genre Gentiana IX. Etude de composés flavoniques et xanthoniques dans les feuilles de *gentiana CampestrisL*. 1ère communication. Helv. Chim. Acta.

[95] Rana V.S., Rawat M.S.M. (2005). A New Xanthone Glycoside and Antioxidant Constituents from the Rhizomes of *Swertia speciosa*. Chem. Biodivers..

[96] Hajimehdipoor H., Dijoux-Franca M.G., Mariotte A.M., Amanzadeh Y., Sadat-Ebrahimi S.E., Ghazi-Khansari M. (2006). Two new xanthone diglycosides from *Swertia longifolia* Boiss. Nat. Prod. Res..

[97] Murray A.P., Faraoni M.B., Castro M.J., Alza N.P., Cavallaro V. (2013). Natural AChE Inhibitors from Plants and their Contribution to Alzheimer’s Disease Therapy. Curr. Neuropharmacol..

[98] Nalivaeva N.N., Turner A.J. (2016). AChE and the amyloid precursor protein (APP)—Cross-talk in Alzheimer’s disease. Chem.-Biol. Interact..

[99] Sherif F., Gottfries C.G., Alafuzoff I., Oreland L. (1992). Brain gamma-aminobutyrate aminotransferase (GABA-T) and monoamine-oxidase (MAO) in patients with alzheimers-disease. J. Neural Transm.-Park. Dis. Dement. Sect..

[100] Urbain A., Marston A., Grilo L.S., Bravo J., Purev O., Purevsuren B., Batsuren D., Reist M., Carrupt P.A., Hostettmann K. (2008). Xanthones from *Gentianella amarella* ssp acuta with acetylcholinesterase and monoamine oxidase inhibitory activities. J. Nat. Prod..

[101] Tang L., Xu X.M., Rinderspacher K.A., Cai C.Q., Ma Y., Long C.L., Feng J.C. (2011). Two new compounds from *Comastoma pedunlulatum*. J. Asian Nat. Prod. Res..

[102] Ding L., Liu B., Zhang S.D., Hou Q., Qi L.L., Zhou Q.Y. (2011). Cytotoxicity, apoptosis-inducing effects and structure-activity relationships of four natural xanthones from *Gentianopsis paludosa* Ma in HepG2 and HL-60 cells. Nat. Prod. Res..

[103] Gao L., Zhou Y., Yan H., Huang F., Wen R., Li G. (2011). Two new xanthone glucosides from *Swertia mussotii* Franch. Heterocycles.

[104] Wan L.S., Min Q.X., Wang Y.L., Yue Y.D., Chen J.C. (2013). Xanthone glycoside constituents of *Swertia kouitchensis* with *α*-glucosidase inhibitory activity. J. Nat. Prod..

[105] Yue Y.D., Zhang Y.T., Liu Z.X., Min Q.X., Wan L.S., Wang Y.L., Xiao Z.Q., Chen J.C. (2014). Xanthone Glycosides from *Swertia bimaculata* with *α*-Glucosidase Inhibitory Activity. Planta Med..

[106] Chen Y.L., Tong Y.F., Wang Q.H. (2014). Two new xanthones from *Lomatogonium carinthiacum*. Chin. J. Nat. Med..

[107] Mahendran G., Manoj M., Murugesh E., Sathish Kumar R., Shanmughavel P., Rajendra Prasad K.J., Narmatha Bai V. (2014). In vivo anti-diabetic, antioxidant and molecular docking studies of 1,2,8-trihydroxy-6-methoxy xanthone and 1,2-dihydroxy-6-methoxyxanthone-8-*O*-*β*-d-xylopyranosyl isolated from *Swertia corymbosa*. Phytomedicine.

[108] Lu S., Tanaka N., Kawazoe K., Murakami K., Damdinjav D., Dorjbal E., Kashiwada Y. (2016). Tetrahydroxanthones from Mongolian medicinal plant *Gentianella amarella* ssp. acuta. J. Nat. Med..

[109] Shi K.L., Wang Y.Q., Jiang Q., Liao Z.X. (2010). Chemical Constituents of *Gentianella azurea*. Chin. J. Nat. Med..

[110] Xu K.P., Li F.S., Liu J.F., Tan J.B., Zhang L.H., Zeng G.Y., Tan G.S. (2008). Studies on Chemical Constituents of *Swertia nervosa* (G.Don) Wall. Chin. Pharm. J..

[111] Tan G.S., Xu P.S., Tian H.Y., Xu K.P., Dai Z.Y. (2000). Studies on the chemical constituents of *Swertia davidi*. Chin. Pharm. J..

[112] Tian C.W., Zhang T.J., Wang L.L., Shan Q., Jiang L.H. (2014). The hepatoprotective effect and chemical constituents of total iridoids and xanthones extracted from *Swertia mussotii* Franch. J. Ethnopharmacol..

[113] Wang Z., Wu G., Liu H., Xing N., Sun Y., Zhai Y., Yang B., Kong A.T., Kuang H., Wang Q. (2017). Cardioprotective effect of the xanthones from *Gentianella acuta* against myocardial ischemia/reperfusion injury in isolated rat heart. Biomed. Pharmacother..

[114] Tosa H., Iinuma M., Murakami K., Ito T., Tanaka T., Chelladurai V., Riswan S. (1997). Three xanthones from *Poeciloneuron pauciflorum* and *Mammea acuminata*. Phytochemistry.

[115] Fu P., Zhang W.D., Liu R.H., Li T.Z., Shen Y.H., Li H.L., Zhang W., Chen H.S. (2006). Two new xanthones from *Hypericum japonicum*. Nat. Prod. Res..

[116] Ishiguro K., Yamamota R., Oku H. (1999). Patulosides A and B, novel xanthone glycosides from cell suspension cultures of *Hypericum patulum*. J. Nat. Prod..

[117] Kitanov G.M., Nedialkov P.T. (2000). Xanthohypericoside, a new xanthone-*O*-glucoside from *Hypericum annulatum*. Pharmazie.

[118] Yan X.T., An Z., Huangfu Y., Zhang Y.T., Li C.H., Chen X., Liu P.L., Gao J.M. (2019). Polycyclic polyprenylated acylphloroglucinol and phenolic metabolites from the aerial parts of *Hypericum elatoides* and their neuroprotective and anti-neuroinflammatory activities. Phytochemistry.

[119] Woo K.W., Lee K.H., Jang J.H., Kim M.S., Cho H.W., Cho J.H., An B. (2016). Anti-inflammatory Constituents from the Aerial Parts of *Iris minutiaurea*. Nat. Prod. Commun..

[120] Li W.K., Chan C.L., Leung H.W., Yeung H.W., Xiao P.G. (1999). Xanthones from *Polygala caudata*. Phytochemistry.

[121] Abd El-Kader A.M., Ahmed A.S., Nafady A.M., Ibraheim Z.Z. (2013). Xanthone and lignan glycosides from the aerial parts of *Polygonum bellardii* all growing in Egypt. Pharmacogn. Mag..

[122] Li J., Jiang Y., Tu P.F. (2005). Xanthone *O*-Glycosides and Benzophenone *O*-Glycosides from the Roots of *Polygala tricornis*. J. Nat. Prod..

[123] Jiang Y., Tu P.F. (2002). Xanthone *O*-glycosides from *Polygala tenuifolia*. Phytochemistry.

[124] He K., Fan L.L., Wu T.T., Du J. (2019). A new xanthone glycoside from *Pyrrosia sheareri*. Nat. Prod. Res..

[125] Rezanka T., Dembitsky V.M. (2003). Identification of acylated xanthone glycosides by liquid chromatography-atmospheric pressure chemical ionization mass spectrometry in positive and negative modes from the lichen *Umbilicaria proboscidea*. J. Chromatogr. A.

[126] Rezanka T., Jachymova J., Dembitsky V.M. (2003). Prenylated xanthone glucosides from Ural’s lichen *Umbilicaria proboscidea*. Phytochemistry.

[127] Eltamany E.E., Abdelmohsen U.R., Ibrahim A.K., Hassanean H.A., Hentschel U., Ahmed S.A. (2014). New antibacterial xanthone from the marine sponge-derived Micrococcus sp. EG45. Bioorg. Med. Chem. Lett..

[128] Yang B.J., Chen G.D., Li Y.J., Hu D., Guo L.D., Xiong P., Gao H. (2016). A New Xanthone Glycoside from the Endolichenic Fungus Sporormiella irregularis. Molecules.

[129] Yoneyama T., Iguchi M., Yoshii K., Elshamy A.I., Ban S., Noji M., Umeyama A. (2021). Xanthone glucoside from an insect pathogenic fungus Conoideocrella luteorostrata NBRC106950. Nat. Prod. Res..

[130] Vermes B., Seligmann O., Wagner H. (1985). Synthesis of xanthone *O*-glycosides. III. synthesis of 1-*O* and 8-*O*-*β*-d-glycosides of 5-*O*-methylbellidifolin and de-*O*-methylbellidifolin. Helv. Chim. Acta.

[131] Hu H.G., Wang M.J., Zhao Q.J., Liao H.L., Cai L.Z., Song Y., Zhang J., Yu S.C., Chen W.S., Liu C.M. (2007). Synthesis of mangiferin derivatives as protein tyrosine phosphatase 1B inhibitors. Chem. Nat. Compd..

[132] Wei X., Liang D., Ning M., Wang Q., Meng X., Li Z. (2014). Semi-synthesis of neomangiferin from mangiferin. Tetrahedron Lett..

[133] Wei X., Liang D., Wang Q., Meng X., Li Z. (2016). Total synthesis of mangiferin, homomangiferin, and neomangiferin. Org. Biomol. Chem..

[134] Zarena A.S., Sankar K.U. (2015). Synthesis of *α*-mangostin-d-glucoside in supercritical carbon dioxide media. J. Food Sci. Technol..

[135] Tuoi T.L., Pandey R.P., Gurung R.B., Dhakal D., Sohng J.K. (2014). Efficient enzymatic systems for synthesis of novel *α*-mangostin glycosides exhibiting antibacterial activity against Gram-positive bacteria. Appl. Microbiol. Biotechnol..

